# CAF-derived exosomal circMPP6 drives ovarian cancer metastasis by coordinating nuclear and cytoplasmic regulation of ADAM22 to activate TGF-β/Smad signaling

**DOI:** 10.7150/ijbs.126013

**Published:** 2026-03-30

**Authors:** Xinyi Wei, Xin Chen, Yan Ren, Mengyan Tu, Shenglong Wu, Tianchen Guo, Simin Xiang, Weiguo Lu, Junfen Xu

**Affiliations:** 1 Department of Gynecologic Oncology, Women's Hospital, Zhejiang University School of Medicine, Hangzhou 310006, Zhejiang, China.; 2 Zhejiang Key Laboratory of Maternal and Infant Health, Women's Hospital, Zhejiang University School of Medicine, Hangzhou 310006, China.; 3 Zhejiang Provincial Clinical Research Center for Gynecology, Hangzhou 310006, Zhejiang, China.

**Keywords:** high-grade serous ovarian cancer, CAFs, exosomal circular RNA, CircMPP6, SFPQ/NONO complex, EEF1A2, ADAM22-TGF-β/Smad signaling

## Abstract

Cancer-associated fibroblasts (CAFs) contribute to the metastatic progression of high-grade serous ovarian cancer (HGSOC), partly through the transfer of regulatory RNAs via exosomes. Here, we identify a circRNA, circMPP6 as a key pro-metastatic factor enriched in CAF-derived exosomes. circMPP6 is upregulated in metastatic HGSOC tissues and is associated with poor prognosis. In HGSOC cells, nuclear circMPP6 interacts with SFPQ and NONO to stabilize ADAM22 mRNA, whereas cytoplasmic circMPP6 binds EEF1A2 to enhance ADAM22 protein expression. Elevated ADAM22 levels activate TGF-β/Smad2/3 signaling via binding to ITGB1, promoting proliferation, migration, and invasion *in vitro* and metastasis *in vivo*. Silencing circMPP6 or disrupting the ADAM22 axis attenuates these oncogenic phenotypes. In CAFs, its loading into exosomes is mediated by hnRNPA2B1, enabling its transfer to adjacent tumor cells. These findings reveal a dual regulatory mechanism by which CAFs-derived exosomal circMPP6 enhances ADAM22 expression and activates pro-metastatic TGF-β signaling in HGSOC. Our study highlights circMPP6 as a potential therapeutic target and critical mediator of stromal-tumor communication in ovarian cancer metastasis.

## Introduction

Ovarian cancer remains one of the deadliest malignancies affecting women worldwide, primarily due to its propensity for early intraperitoneal dissemination and metastatic spread at diagnosis, leading to poor clinical outcomes 1. In 2022, over 320,000 new cases and approximately 200,000 deaths were reported globally, highlighting the urgent need for improved therapeutic strategies 2. Among ovarian cancer subtypes, high-grade serous ovarian carcinoma (HGSOC) accounts for the majority of cases and mortality 3, and is characterized by rapid and widespread metastasis within the abdominal cavity, particularly to the omentum 4. Despite advances in surgery and chemotherapy, the survival rates remain poor, necessitating a deeper understanding of the molecular mechanisms underlying HGSOC progression and metastasis.

Increasing evidence has highlighted the pivotal role of the tumor microenvironment (TME) in cancer progression, where stromal components actively communicate with tumor cells to modulate malignancy 5. Cancer-associated fibroblasts (CAFs), a predominant stromal cell type within the TME, promote tumor growth, invasion, and metastasis bysecreting extracellular matrix components, soluble factors, and extracellular vesicles, such as exosomes 6-8. Exosomes, which are nanoscale vesicles (30-150 nm), are emerging as critical mediators of intercellular signaling, transporting proteins, metabolites, and regulatory RNAs, including non-coding RNAs (ncRNAs), between cells 9-12. Among these, circular RNAs (circRNAs), characterized by a covalently closed loop structure that confers remarkable stability, have gained attention as versatile regulators of gene expression with high tissue specificity 13-16, 17. CAF-derived exosomal circRNAs have been implicated in reshaping cancer cell behavior by functioning as miRNA sponges, scaffolding RNA-protein interactions, or modulating transcriptional and post-transcriptional regulation 15, 18, 19. However, the specific contribution of CAF-derived exosomal circRNAs to HGSOC metastasis remains largely unknown.

Here, we report the identification of a novel CAF-enriched circRNA, circMPP6, which is significantly upregulated in metastatic HGSOC tissues relative to primary tumors, with higher expression correlated with poorer patient prognosis. Mechanistically, circMPP6 exerts a dual regulatory role within tumor cells: in the nucleus, it associates with the SFPQ-NONO complex to stabilize ADAM22 mRNA, whereas in the cytoplasm, it binds EEF1A2 to enhance ADAM22 protein expression. This coordinated upregulation of ADAM22 interacts with ITGB1 and activates the TGF-β/Smad2/3 signaling pathway, driving proliferation, migration, invasion, and metastasis of HGSOC cells. Silencing of circMPP6 or disruption of the ADAM22 axis markedly attenuated these malignant phenotypes *in vitro* and* in vivo*. Additionally, we demonstrated that circMPP6 was selectively packaged into CAF-derived exosomes via interaction with the RNA-binding protein hnRNPA2B1 and subsequently transferred to HGSOC cells. Our findings reveal a previously unrecognized mechanism of stromal-tumor communication mediated by CAF-derived exosomal circMPP6, highlighting its critical oncogenic role in HGSOC metastasis and establishing circMPP6 as a promising therapeutic target for ovarian cancer intervention.

## Results

### circMPP6 is highly upregulated in metastatic ovarian cancer and correlates with poor prognosis

**Figure 1:**
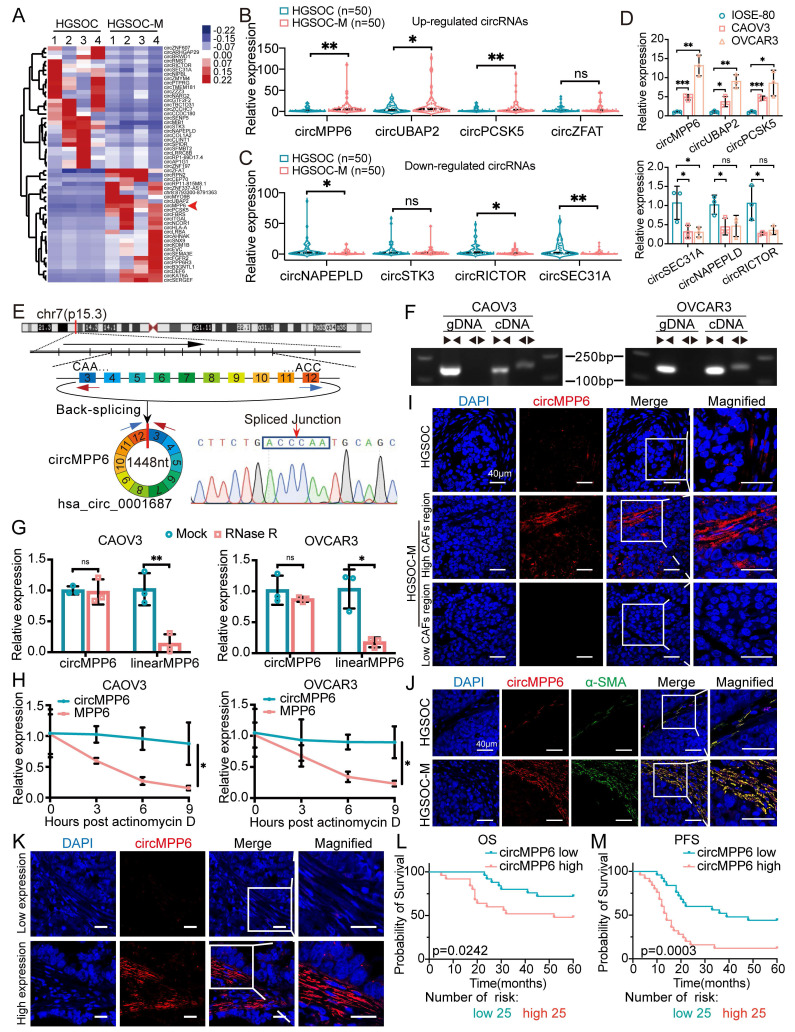
circMPP6 is upregulated in ovarian cancer and correlates with poor prognosis. (**A**) heatmap of differentially expressed circRNAs in 4 pairs of HGSOC omental metastasis tissues (HGSOC-M) and their corresponding primary tissues (HGSOC) by high-throughput sequencing. Red arrow indicates circMPP6. (**B-C**) RT-qPCR analysis of 4 up-regulated circRNAs (B) and 4 down-regulated circRNAs (C) in 50 pairs of HGSOC-M and HGSOC tissues. (**D**) circRNAs expression levels in IOSE-80 and two HGSOC cell lines measured by qRT-PCR. (**E**) Schematic of the MPP6 gene and transcript structure. circMPP6 is formed from exons 3-12 of MPP6 via back-splicing; the back-splice junction was confirmed by Sanger sequencing. (**F**) PCR and agarose gel electrophoresis of circMPP6 using divergent and convergent primers in cDNA or gDNA from CAOV3 and OVCAR3 cells. (**G**) Relative RNA levels analyzed by RT-qPCR after RNase R treatment or mock treatment in CAOV3 and OVCAR3 cells. (**H**) RT-qPCR of circMPP6 and linear MPP6 abundance in CAOV3 and OVCAR3 cells treated with actinomycin D (5 μg/ml) at indicated times. (**I**) FISH analysis of circMPP6 expression and localization in primary and metastatic HGSOC tissues. Red: circMPP6 labeled with Cy3 probe; blue: nuclei stained with DAPI. (**J**) FISH analysis of circMPP6 and immunofluorescence analysis of α-SMA expression in HGSOC and HGSOC-M tissues. **(K)** Representative FISH images of circMPP6 in HGSOC tissues. **(L-M)** Kaplan-Meier survival analysis of overall (L) and progression-free survival (M) in 50 HGSOC patients with differing circMPP6 expression levels. Data are representative of three independent experiments. *p < 0.05, **p < 0.01, ***p < 0.001, ****p < 0.0001.

To identify critical circRNAs involved in omental metastasis of HGSOC, high-throughput sequencing was performed on four pairs of HGSOC omental metastatic tissues and their corresponding primary tissues. The results revealed 54 differentially expressed circRNAs, including 26 upregulated and 28 downregulated circRNAs, in omental metastases compared with primary HGSOC tissues (Fig. 1A). Briefly, four most upregulated circRNAs (circMPP6, circUBAP2, circPCSK5, and circZFAT) and four most downregulated circRNAs (circNAPEPLD, circSTK3, circRICTOR, and circSEC31A) identified from the initial screening were validated in 50 pairs HGSOC primary and omental metastatic tissues (Fig. 1B and C). Six candidates showed significant differential expression, among which circMPP6 displayed the lowest P-value (0.0017). Expanded validation in HGSOC cell lines (CAOV3, OVCAR3) and the normal ovarian epithelial line (IOSE-80) further confirmed that circMPP6 exhibited both the strongest statistical significance (P < 0.001) and the largest fold change (Fig. 1D), supporting its potential role in metastasis.

Bioinformatics analysis indicated that circMPP6 is derived from exons 3 to 12 of MPP6 and has a sequence length of 1448 nucleotides. This was experimentally confirmed by RT-PCR with divergent primers and Sanger sequencing (Fig.1E), and aligned with the annotation in CircBase (http://www.circbase.org/). Divergent primers amplified circMPP6 from cDNA but not from genomic DNA (Fig. 1F), ruling out genomic rearrangements or PCR artifacts as its origin. Further analysis revealed that circMPP6 was resistant to RNase R digestion (Fig. 1G) and actinomycin-D treatment (Fig. 1H), confirming its circular structure.

Using FISH assays on paraffin-embedded samples, we observed a significantly higher circMPP6 expression in omental metastatic tissues than in primary HGSOC tissues (Fig. 1I). Notably, the localization of circMPP6 was predominantly observed in the stromal regions adjacent to the tumor regions, with enriched expression also found in tumor cells near the stroma and minimal expression in tumor cells distant from the stroma (Fig. 1I and Supplementary Fig. S1A). The results from FISH-IF assays demonstrated co-localization with α-SMA, a CAF marker, indicating that circMPP6 expression in the stroma was predominantly located in areas with high CAF infiltration (Fig. 1J). A retrospective clinicopathological analysis of another 50 patients with HGSOC revealed that higher circMPP6 levels in CAFs were associated with shorter overall survival (Fig. 1K and L) and progression-free survival (Fig. 1M), highlighting circMPP6 as a potential prognostic biomarker for HGSOC.

### CAF-specific circRNA circMPP6 promotes ovarian cancer progression

**Figure 2:**
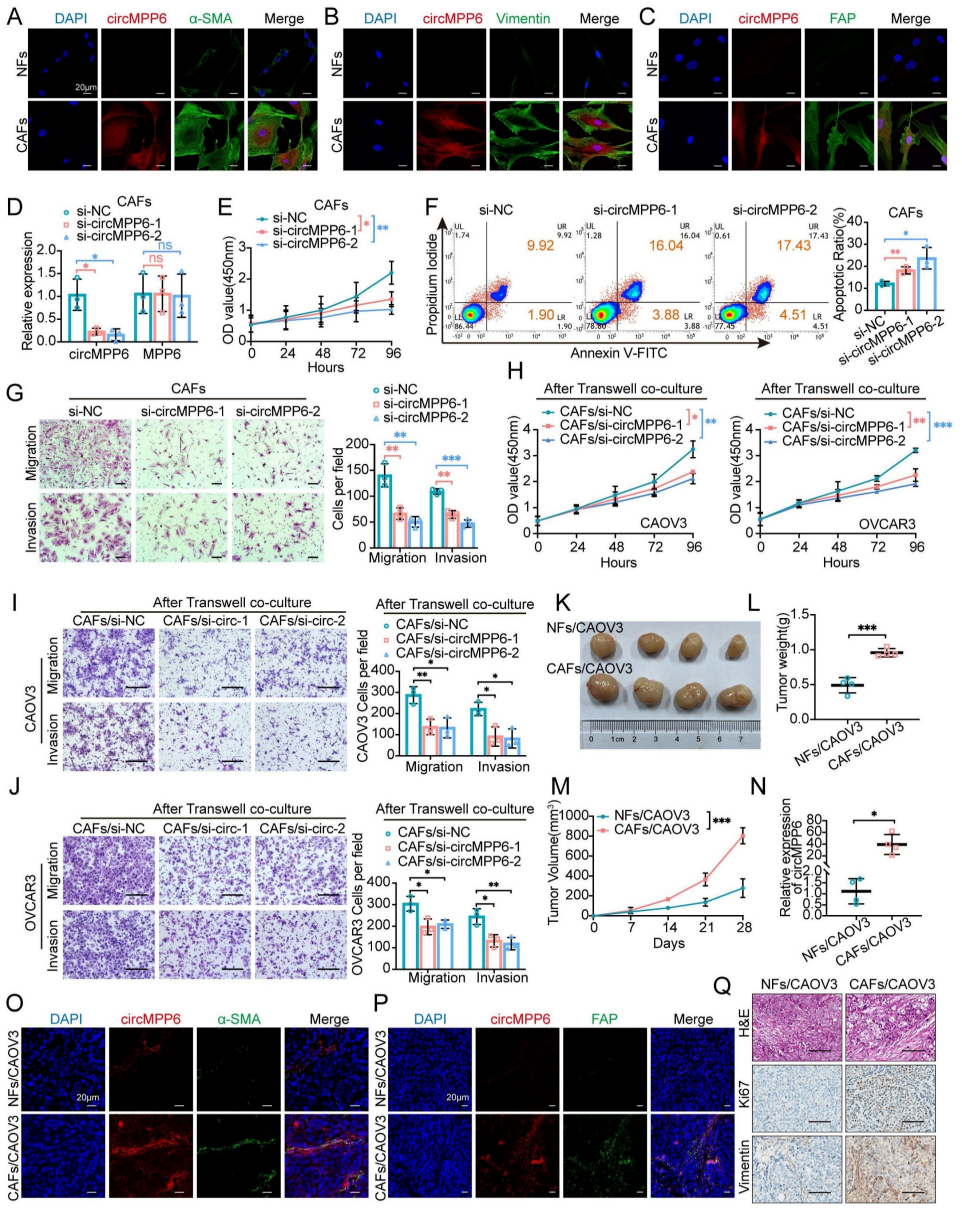
Validation and function of CAF-specific circRNA circMPP6. (**A-C**) FISH analysis of circMPP6 (red) and α-SMA (A), Vimentin (B), or FAP (C) immunofluorescence (green) in NFs and CAFs. Scale bar: 20 μm. (**D**) Expression levels of circMPP6 and MPP6 mRNA in CAFs after transfection with circMPP6-specific siRNAs or control siRNA analyzed by RT-qPCR. (**E**) Cell proliferation in CAFs measured by CCK-8 assay. (**F**) Apoptosis in CAFs determined by flow cytometry. (**G**) CAF migration and invasion measured by Transwell assay. (**H**) Changes in CAOV3 (left) or OVCAR3 (right) proliferation after co-culture with CAFs with or without circMPP6 knockdown. (**I-J**) Migration and invasion of CAOV3 (I) or OVCAR3 (J) co-cultured with CAFs with or without circMPP6 knockdown. Scale bar: 100 mm. (**K-N**) Images of tumors (K), tumor weights (L), and tumor growth curves (M), and circMPP6 expression levels (N) in xenografts from CAOV3 cells co-transplanted with NFs or CAFs. (**O-P**) FISH analysis of circMPP6 (red) and immunofluorescence staining (green) of α-SMA (O) or FAP (P) in xenografts. Scale bar: 20 μm. (**Q**) H&E and immunohistochemical analysis of Ki67 and Vimentin in xenografts. *p < 0.05; **p < 0.01; ***p < 0.001; ns, not significant. Data are mean ± SD from three independent experiments.

To confirm the source of circMPP6, we isolated CAFs and normal fibroblasts (NFs) from the HGSOC metastatic and normal omental tissues, respectively. While expressing the activation markers α-SMA, Vimentin, and FAP, CAFs showed dramatically higher circMPP6 expression (Fig. 2A-C). To assess the function of circMPP6 in ovarian cancer, we transfected CAFs with circMPP6-specific siRNAs, which effectively reduced circMPP6 expression without altering the linear MPP6 mRNA levels (Fig. 2D). Downregulation of circMPP6 in CAFs inhibited their proliferation, migration, and invasion, while promoting apoptosis *in vitro* (Fig. 2E-G). Furthermore, ovarian cancer cell lines (CAOV3 and OVCAR3) co-cultured with circMPP6-knockdown CAFs showed reduced proliferation, migration and invasion compared to those co-cultured with control CAFs (Fig. 2H-J).

*In vivo*, CAFs promoted tumor growth in a mouse subcutaneous xenograft model. Xenografts formed from CAOV3 cells with CAFs exhibited significantly faster growth than those formed with NFs (Fig. 2K-M). Consistent with *in vitro* findings, these xenografts showed elevated circMPP6 levels (Fig. 2N), and FISH-IF confirmed the increased expression of α-SMA (Fig. 2O) and FAP (Fig. 2P) in the CAF-associated xenografts. Hematoxylin and eosin (H&E) staining, along with Ki67 and Vimentin analyses, revealed higher cell proliferation and metastatic potential in xenografts co-transplanted with CAFs (Fig. 2Q and Supplementary Fig. S1B-C). Collectively, these findings suggest that CAF-derived circMPP6 plays an oncogenic role in ovarian cancer progression, both *in vitro* and *in vivo*.

### Transfer of circMPP6 from CAFs to HGSOC cells via exosomes

**Figure 3:**
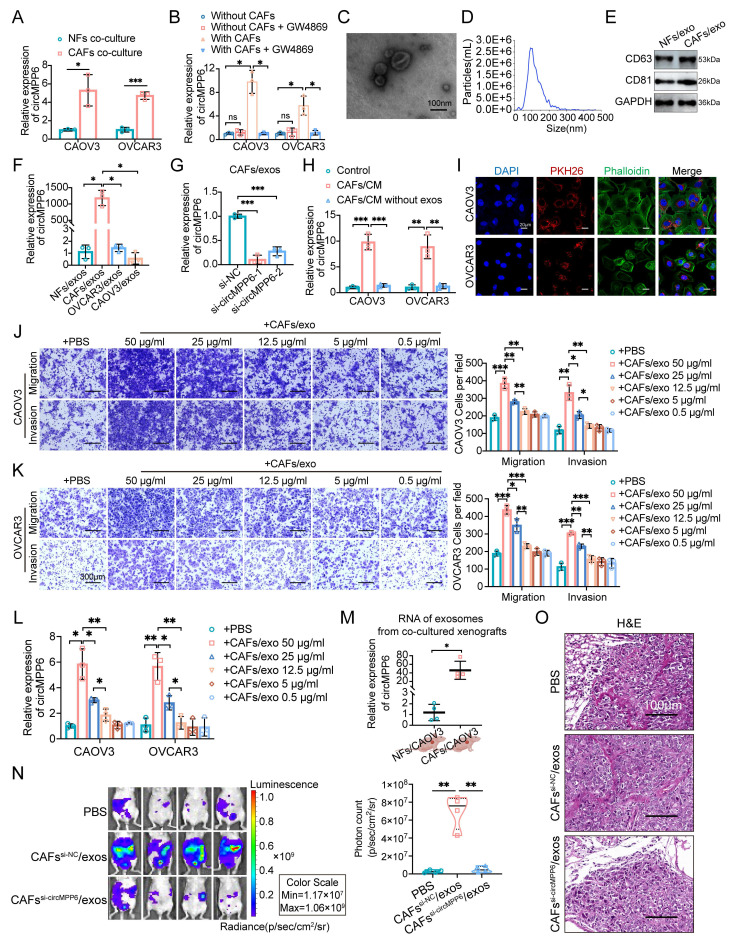
Exosomal transfer of circMPP6 from CAFs to HGSOC cells. (**A**) circMPP6 expression in CAOV3 and OVCAR3 cells co-cultured with CAFs or NFs by RT-qPCR. (**B**) circMPP6 levels in CAOV3 and OVCAR3 cells co-cultured with CAFs with or without GW4869 (10μM). (**C**) Representative transmission electron microscopy images of exosomes purified from cell supernatant. Scale bar, 100 nm. (**D**). Size distribution of exosomes measured by nanoparticle analysis. (**E**). Western blot analysis of CD63, CD81 in the exosomes fraction. (**F**) RT-qPCR of circMPP6 in exosomes from NFs, CAFs, CAOV3, and OVCAR3 cells. (**G**) RT-qPCR of circMPP6 in exosomes from CAFs transfected with circMPP6-specific siRNAs or control. (**H**) RT-qPCR of circMPP6 in CAOV3 and OVCAR3 cells after co-cultured with CAFs conditioned medium with or without exosomes. (**I**) PKH26-labeled CAFs exosomes (red) incubated with CAOV3 or OVCAR3 cells observed by confocal microscopy. Scale bar: 20 μm. (**J-K**) Migration and invasion of CAOV3 cells (J) and OVCAR3 cells (K) under different conditions analyzed by Transwell assay. Scale bar: 300 μm. (**L**) RT-qPCR of circMPP6 in CAOV3 or OVCAR3 cells under different conditions. (**M**) RT-qPCR of circMPP6 in exosomes from xenograft tumors co-transplanted with CAOV3 cells and NFs or CAFs. (**N**) Left: Representative bioluminescence of nude mice with intraperitoneal xenograft. Right: Photon count indicating tumor burden. (**O**) H&E staining in metastatic tumors. Scale bar: 100 μm. *p < 0.05; **p < 0.01; ***p < 0.001; ns, not significant. Data are mean ± SD from three independent experiments.

Our findings revealed that in the co-culture system, CAFs significantly increased circMPP6 expression levels in CAOV3 and OVCAR3 ovarian cancer cells compared to those co-cultured with NFs (Fig. 3A), indicating that CAFs may transfer circMPP6 to ovarian cancer cells via the extracellular environment. Considering the known role of exosomes in intercellular communication, we hypothesized that circMPP6 could be packaged into exosomes. To investigate this, the exosome secretion inhibitor GW4869 was applied to a Transwell co-culture system. qRT-PCR analysis demonstrated elevated circMPP6 levels in ovarian cancer cells co-cultured with CAFs, and this increase was markedly abolished by GW4869 treatment, indicating that circMPP6 was enclosed in exosomes (Fig. 3B).

Exosomes were isolated by ultracentrifugation, and transmission electron microscopy confirmed the presence of membrane-bound spherical vesicles ranging from 30 to 150 nm in size (Fig. 3C), with a predominant size of approximately 100 nm (Fig. 3D). Western blotting and high-sensitivity flow cytometry analyses identified the exosome-specific markers CD63 and CD81 (Fig. 3E and Supplementary Fig. S1D). To further substantiate exosome-mediated transfer of circMPP6 to tumor cells, we compared circMPP6 expression in NFs, CAFs, CAOV3 and OVCAR3 cells (Supplementary Fig.S1E), showing that circMPP6 is markedly enriched in CAFs and further concentrated in CAF-derived exosomes (Fig. 3F), providing the rationale for focusing on tumor microenvironment-mediated transfer. Moreover, when circMPP6 was knocked down in CAFs, its levels in the CAF-derived exosomes were notably reduced (Fig. 3G). Next, conditioned media (CM) from CAFs, with or without exosome depletion, was applied to CAOV3 and OVCAR3 recipient cells. Exosome-depleted CM failed to elevate circMPP6 levels, confirming that exosomes are the primary mediators of circMPP6 transfer (Fig 3H). PKH26 is a red fluorescent dye used to label cell membranes and track extracellular vesicles 20. Phalloidin binds F-actin and is used to visualize the cytoskeleton. To investigate whether exosomes could be transmitted between cells, exosomes of CAFs were labeled with PKH26 and incubated with CAOV3 and OVCAR3 cells for 24 h. PKH26 staining indicated that CAF-derived exosomes could fuse with and be taken up by ovarian cancer cells (CAOV3 and OVCAR3), thereby transferring circMPP6 (Fig. 3I).

CAF-derived exosomes also promoted the migration and invasion of CAOV3 and OVCAR3 cells, and this effect increased in proportion to the concentration of CAF-derived exosomes (Fig. 3J and K). Additionally, circMPP6 levels in CAOV3 and OVCAR3 cells increased significantly when co-cultured with CAF-derived exosomes, and circMPP6 levels varied according to the concentration of CAF-derived exosomes (Fig. 3L). Consistent with the *in vitro* experiments, we found that exosomes isolated from CAOV3 xenografts co-implanted with CAFs showed higher circMPP6 expression than those from xenografts co-implanted with NFs (Fig. 3M). Furthermore, *in vivo* abdominal metastatic models were established, which showed that intraperitoneal injection of CAF-derived exosomes markedly enhanced the metastatic potential of ovarian cancer cells, whereas this effect was substantially diminished by circMPP6-knockdown exosomes (Fig. 3N). Importantly, no significant differences in body weight were observed between the groups (Supplementary Fig. S1F). H&E staining confirmed the typical cancer morphology in the xenograft tissues (Fig. 3O), with no visible damage to the major organs (Supplementary Fig. S1G).

### circMPP6 enhances proliferation, migration, and invasion in HGSOC cells

**Figure 4:**
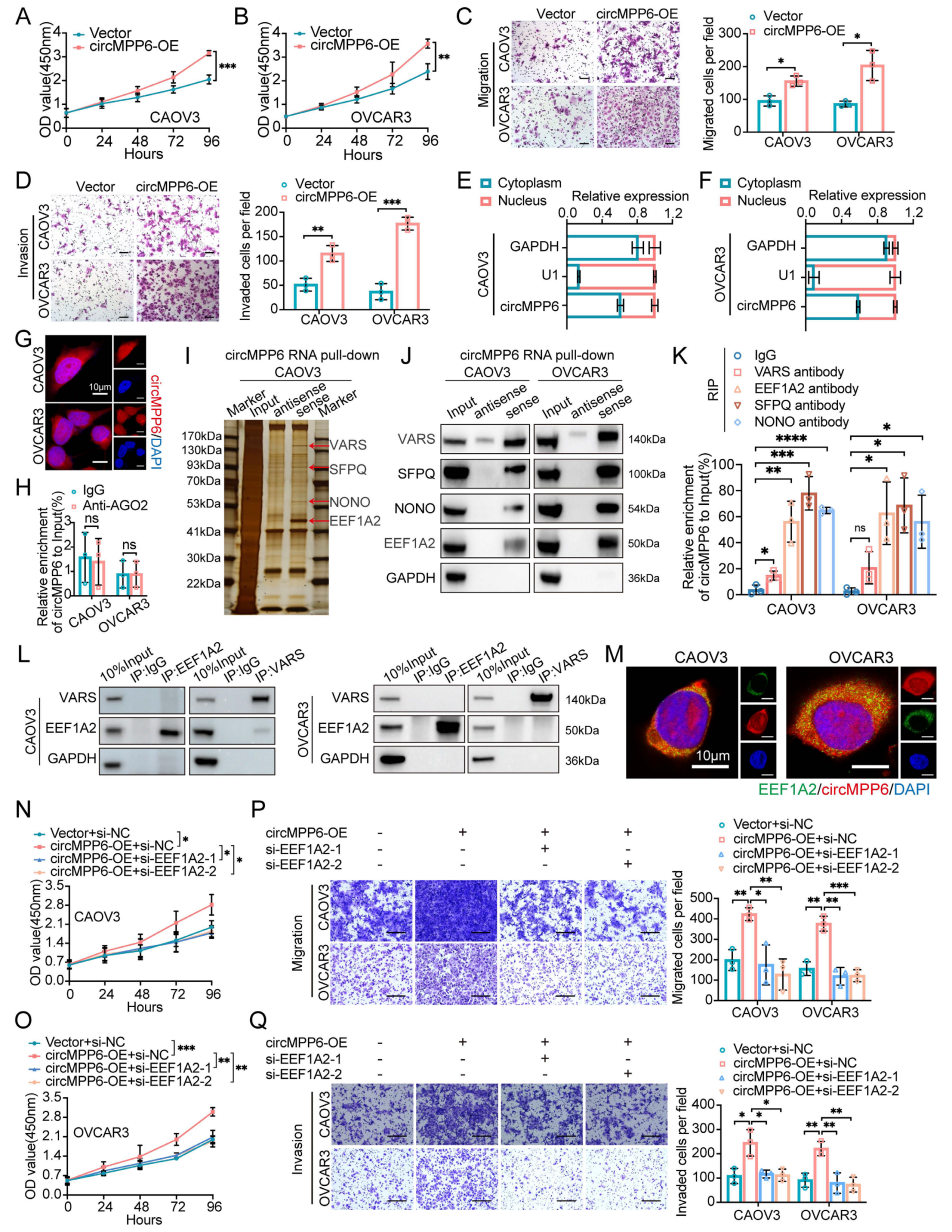
circMPP6 promotes ovarian cancer proliferation, migration, and invasion via interaction with RBPs. (**A-B**) Proliferation in CAOV3(A) and OVCAR3(B)cells transfected with circMPP6 plasmid or control plasmid by CCK-8. (**C-D**) Migration (C) and invasion (D) assays for HGSOC cells transfected with circMPP6 or control plasmid. (**E-F**) The distribution of circMPP6 was detected by qRT-PCR in CAOV3 (**E**) and OVCAR3 (**F**) cells. U1 and GAPDH were used as nuclear and cytoplasmic positive controls, respectively. (**G**) circMPP6 cellular distribution by FISH. Red: circMPP6 with Cy3 probe; blue: DAPI-stained nuclei. Scale bar: 10 μM. (**H**) RNA immunoprecipitation of circMPP6-AGO2 interaction. (**I**) RNA pull-down with biotin-labeled circMPP6 probes; sliver staining and mass spectrometry of pulled proteins. (**J**) Western blot validation of pulled proteins with biotin-labeled circMPP6 probes. GAPDH as loading control. (K) RNA immunoprecipitation in CAOV3 and OVCAR3 using specific antibodies. (**L**) Co-IP of VARS and EEF1A2 interaction. (**M**) RNA-FISH for cirMPP6 (red) and immunofluorescence for EEF1A2 (green) in CAOV3 and OVCAR3. Scale bar: 10 μm. (**N-R**) HGSOC cells transfected with circMPP6 plasmid, si-EEF1A2-1, si-EEF1A2-2, or controls; analyzed for viability (N-O), migration (P), and invasion (Q).

To elucidate the functional role of circMPP6 in HGSOC cells, circMPP6 expression was manipulated using RNA interference and overexpression plasmids in CAOV3 and OVCAR3 cells. Knockdown experiments showed efficient circMPP6 suppression without affecting linear MPP6 mRNA expression (Supplementary Fig. S2A-B). The CCK-8 assay revealed a significant reduction in cell proliferation following circMPP6 downregulation (Supplementary Fig. S2C-D). Furthermore, circMPP6 knockdown inhibited migration and invasion capabilities in HGSOC cells (Supplementary Fig. S2E-F). Overexpression assays confirmed successful upregulation of circMPP6 (Supplementary Fig. S2G). However, circMPP6 overexpression had the opposite effect, enhancing these oncogenic behaviors (Fig. 4A-D). Collectively, these findings suggest that circMPP6 acts as an oncogene, promoting the proliferation, migration, and invasion of HGSOC cells.

### circMPP6 directly interacts with EEF1A2, SFPQ, and NONO in HGSOC cells

Given the role of circMPP6 in promoting ovarian cancer progression, we sought to elucidate its underlying mechanisms in ovarian cancer cells. We began by determining the subcellular localization of circMPP6 using cytoplasmic/nuclear fractionation analysis (Fig.4E-F) and a FISH assay (Fig. 4G), which revealed that circMPP6 was distributed in both the cytoplasm and nucleus of CAOV3 and OVCAR3 cells. Although circRNAs often function through a ceRNA mechanism, our initial hypothesis that cytoplasmic circMPP6 acts as a miRNA sponge was disproved when circMPP6 did not bind to AGO2 (Fig. 4H).

We conducted a mass spectrometry (MS) analysis after RNA pull-down to identify the proteins associated with circMPP6 (Fig. 4I and Supplementary Fig. S2H). The results revealed that four proteins were significantly enriched in circMPP6: VARS, EEF1A2, SFPQ, and NONO (Fig. 4J). RNA-binding protein immunoprecipitation (RIP) assays using antibodies against these proteins confirmed that circMPP6 binds strongly to EEF1A2, SFPQ, and NONO, but exhibits relatively weaker specificity for VARS (Fig. 4K). Although both VARS and EEF1A2 are known translation elongation factors 21, co-IP assays indicated no interaction between them in CAOV3 and OVCAR3 cells (Fig. 4L). EEF1A2 has been reported to be upregulated in various cancers 22, and accumulating evidence has elucidated its oncogenic mechanisms, including its interaction with circRNAs or lncRNAs 23, 24, which further promote cancer progression.

RNA-FISH and IF assays showed that circMPP6 co-localized with EEF1A2 in the cytoplasm of CAOV3 and OVCAR3 cells (Fig. 4M). To clarify EEF1A2's role, we established EEF1A2-knockdown cell models (Supplementary Fig. S3A-B). The knockdown of EEF1A2 inhibited cell proliferation, migration, and invasion (Supplementary Fig. S3C-F). Furthermore, the enhanced phenotypes caused by circMPP6 overexpression were abrogated in EEF1A2-knockdown cells (Fig. 4N-Q), indicating that circMPP6 promotes oncogenic processes in HGSOC cells via EEF1A2 in the cytoplasm.

### circMPP6-SFPQ-NONO interaction in the nucleus enhances ovarian cancer cell proliferation, migration, and invasion

**Figure 5:**
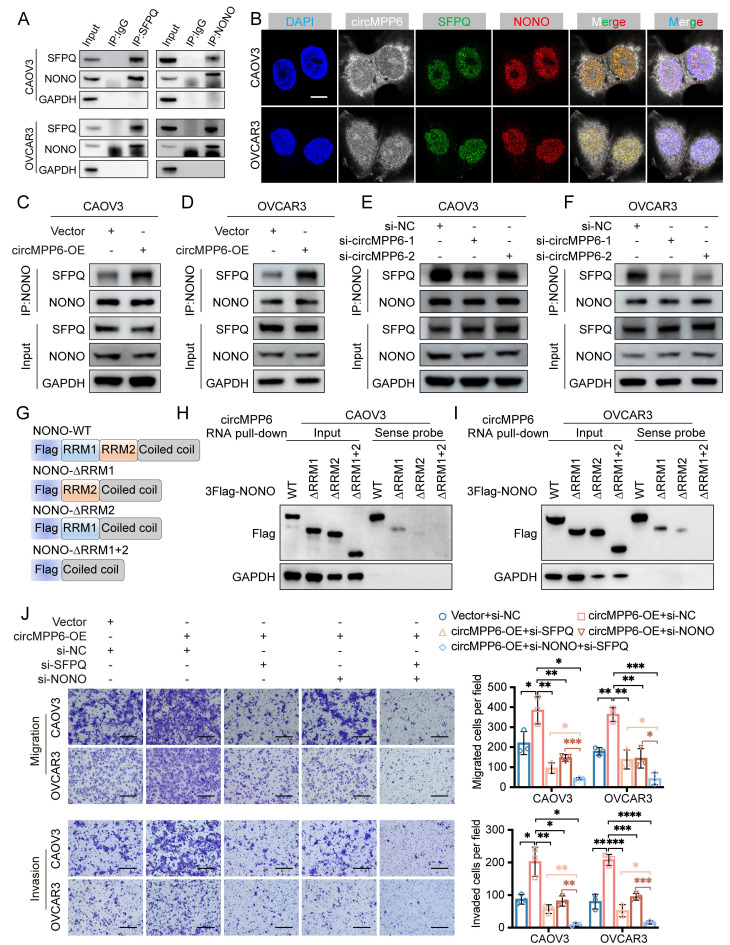
circMPP6 enhances SFPQ and NONO interaction in ovarian cancer cells. (**A**) Co-IP analysis of NONO and SFPQ interactions. (**B**) RNA-FISH of cirMPP6 (white), immunofluorescence of SFPQ (green) and NONO (red) in CAOV3 and OVCAR3 cells. (**C-D**) Co-IP of NONO-SFPQ in CAOV3 (C) and OVCAR3 (D) cells after circMPP6 or a control transfection. (**E-F**) Co-IP of NONO-SFPQ in CAOV3 (E) and OVCAR3 (F) cells after circMPP6 knockdown or control. (**G**) Schematic of NONO domain structure. (**H-I**) RNA pull-down depicting circMPP6-NONO interaction by western blot after recombinant NONO protein incubation. (**J**) Migration and invasion assays of HGSOC cells transfected with circMPP6 plasmid, si-SFPQ, si-NONO, or controls.

SFPQ and NONO are core components of the paraspeckle 25, and both are multifunctional RNA-binding proteins widely involved in RNA splicing, transport, stability, RNA metabolism processing and transcriptional regulation, thereby participating in RNA-mediated tumorigenesis and metastasis 26-28. Importantly, the formation of the SFPQ-NONO heterodimer has been shown to exert a reinforcing effect on the functional activity of either SFPQ or NONO when they act alone 29. Previous studies have revealed that circRNAs could function as either tumor promoters or suppressors by regulating the formation of the NONO-SFPQ protein complex 30, 31. Further experiments confirmed that SFPQ and NONO co-immunoprecipitated in HGSOC cells (Fig. 5A), and RNA-FISH and IF assays demonstrated partial co-localization of circMPP6, SFPQ, and NONO in the nucleus (Fig. 5B). Despite circMPP6 binding to both proteins, changes in circMPP6 expression did not alter SFPQ and NONO levels (Fig. 5C-F). However, circMPP6 enhanced the stability of the SFPQ-NONO interaction, which was reduced by circMPP6 knockdown (Fig. 5C-F). Knockdown and co-IP assays (Supplementary Figure S4A-B) show that circMPP6 binding to SFPQ largely depends on NONO, while circMPP6 interaction with NONO remains after SFPQ knockdown. These results indicate that circMPP6 primarily binds directly to NONO, with only a weaker direct interaction with SFPQ. Truncated NONO variants were generated to map the interaction sites of NONO. NONO is an RNA-binding protein containing two RNA recognition motifs (RRMs). We constructed a 3×FLAG-tagged full-length NONO protein along with three other NONO variants with truncated RRM domains (Fig. 5G). After transfecting CAOV3 and OVCAR3 cells with these plasmids, we evaluated their ability to bind to circMPP6 using FLAG immunoblotting following an RNA pull-down assay. The results showed that NONO variants with both RRM1 (74-141 amino acids [aa]) and RRM2(148-229aa) truncated completely or with only RRM2 (148-229aa) truncated largely lost the ability to bind circMPP6 (Fig. 5H-I).In contrast, the NONO variant with only RRM1 truncated (74-141aa) still retained some binding ability (Fig. 5H-I), suggesting that RRM2 may play a primary role in NONO's interaction with circMPP6, with RRM1 contributing to a lesser extent.

Additionally, we observed that the increased proliferation, migration, and invasion abilities induced by circMPP6 overexpression in CAOV3 and OVCAR3 cells were partially reversed by the knockdown of either SFPQ or NONO, and completely reversed by the combined knockdown of both SFPQ and NONO (Fig. 5J, Supplementary Fig. S4C-D).

### circMPP6 modulates ADAM22 stability through SFPQ and NONO in ovarian cancer cells

**Figure 6:**
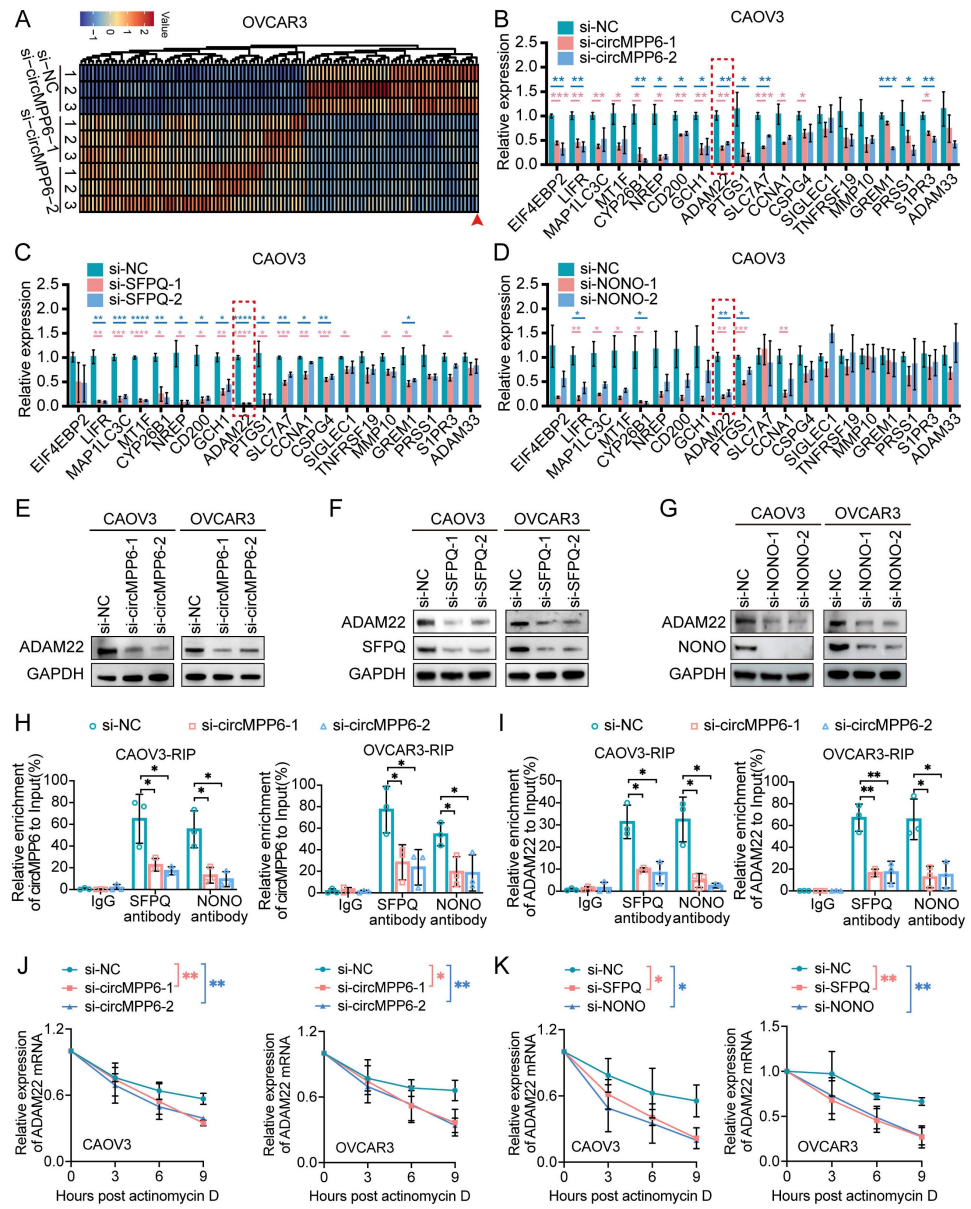
ADAM22 is positively correlated with circMPP6 expression. (**A**) Heatmap of differentially expressed mRNAs in circMPP6 knockdown versus control OVCAR3 cells (**B**) RT-qPCR of differentially expressed mRNAs in CAOV3 with circMPP6 knockdown. (**C-D**) RT-qPCR of mRNAs in CAOV3 with SFPQ (C) or NONO (D) knockdown. (**E-G**) ADAM22 protein levels by western blot after circMPP6 (**E**), SFPQ (**F**) or NONO (**G**) knockdown in CAOV3 and OVCAR3 cells. (**H-I**) circMPP6 (H) and ADAM22 mRNA (I) recruited by SFPQ or NONO antibodies with or without circMPP6 knockdown by RIP in CAOV3 and OVCAR3 cells. (**J**) ADAM22 mRNA expression in circMPP6 knockdown CAOV3 and OVCAR3 cells treated with actinomycin D. (**K**) ADAM22 mRNA expression in NONO or SFPQ knockdown CAOV3 and OVCAR3 cells treated with actinomycin D. *p < 0.05, **p < 0.01, ***p < 0.001, ****p < 0.0001.

To explore the mechanism through which circMPP6 facilitates ovarian cancer metastasis, we performed transcriptome sequencing of OVCAR3 cells with and without circMPP6 knockdown. A total of 104 differentially expressed mRNAs were identified (|log_2_ fold change| > 1, p < 0.05), comprising 59 upregulated and 45 downregulated genes, following circMPP6 knockdown (Fig. 6A). Among these, 20 downregulated genes were selected for validation in CAOV3 and OVCAR3 cells after circMPP6, SFPQ, or NONO knockdown. Notably, ADAM22 was consistently downregulated in both cell lines upon the knockdown of circMPP6, SFPQ, and NONO (Fig. 6B-D, Supplementary Fig. S5A-C). Additionally, ADAM22 protein levels were reduced following the knockdown of circMPP6, SFPQ, or NONO in both CAOV3 and OVCAR3 cells (Fig. 6E-G).

We further validated the interactions between SFPQ and ADAM22 mRNA, as well as NONO and ADAM22 mRNA (Supplementary Fig. S5D), suggesting that SFPQ and NONO may play key roles in circMPP6-mediated regulation of ADAM22 expression. RIP assays demonstrated that knockdown of circMPP6 reduced the recruitment of both circMPP6 and ADAM22 mRNA by SFPQ and NONO in CAOV3 and OVCAR3 cells (Fig. 6H-I). Importantly, circMPP6 knockdown significantly decreased the stability of ADAM22 mRNA (Fig. 6J), with similar destabilizing effects observed after SFPQ and NONO knockdown (Fig. 6K). Overall, our findings suggest that circMPP6 interacts with SFPQ and NONO proteins, stabilizing the SFPQ-NONO complex, and consequently enhancing ADAM22 mRNA stability in ovarian cancer cells. We also observed interactions between EEF1A2 and ADAM22 mRNA (Supplementary Fig. S5E) and EEF1A2 knockdown reduced ADAM22 protein levels (Supplementary Fig. S5F), indicating that ADAM22 expression was regulated by both circMPP6 and EEF1A2. Similarly, EEF1A2 knockdown significantly decreased the stability of ADAM22 mRNA (Supplementary Fig. S5G-H), providing direct evidence that the circMPP6-EEF1A2 axis stabilizes ADAM22 transcripts.

### ADAM22 mediates the role of circMPP6 in enhancing ovarian cancer cell proliferation, migration, and invasion and activating TGF-β/Smad signaling

**Figure 7:**
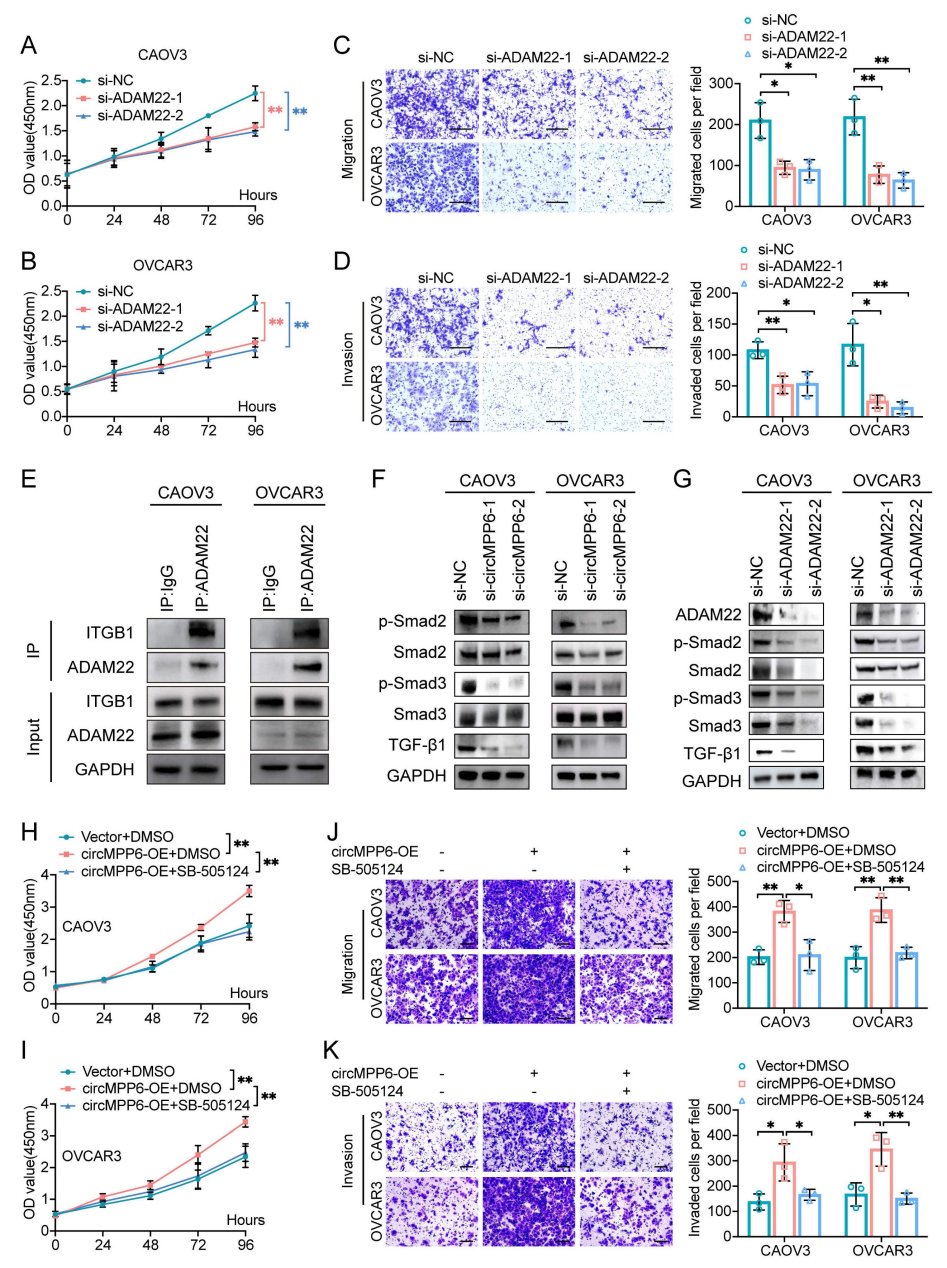
ADAM22 mediates circMPP6 effects on HGSOC cell viability and metastasis and activates TGF-β/Smad signaling. (**A-B**) CAOV3 cells (**A**) and OVCAR3 cells (**B**) transfected with ADAM22 siRNAs or control analyzed for proliferation by CCK-8. (**C-D**) Migration (C) and invasion (D) assays for HGSOC cells transfected with circMPP6 siRNAs or control. (**E**) Co-IP analysis of ADAM22 and ITGB1 interactions. (**F-G**) Western blot analysis of Smad2, p-Smad2, Smad3, p-Smad3, TGF-β after knockdown of circMPP6 (F) or AMAM22 (G). (**H-I**) Effect of SB505124 on CAOV3 and OVCAR3 cell proliferation upon circMPP6 overexpression measured using CCK-8 assay. (**J-K**) Effect of SB505124 on CAOV3 and OVCAR3 cell migration (J) and invasion (K) upon circMPP6 overexpression measured using Transwell assays. *p < 0.05, **p < 0.01, ***p < 0.001.

To investigate ADAM22's functional role, we established ADAM22-knockdown models using two siRNAs (Supplementary Fig. S6A-B). Our experiments revealed that ADAM22 knockdown significantly inhibited the proliferation, migration, and invasion of ovarian cancer cells (Fig. 7A-D).

ADAM22 promotes cell proliferation and invasion by binding to integrins in pituitary adenoma 32. Endogenous co-immunoprecipitation assay confirmed that ADAM22 forms a complex with ITGB1 in ovarian cancer cells (Fig. 7E). Integrin proteins, involved in a variety of physiological and pathological processes, including cell survival, proliferation, migration, and invasion, were verified to activate TGF-β signaling 33-35. Considering that circMPP6/ADAM22 mainly promotes cell proliferation, migration, and invasion in HGSOC, we detected downstream molecules of the TGF-β pathway associated with these processes. The effects of circMPP6 on the level of TGF-β in CAOV3 and OVCAR3 were detected by Western blot (Fig. 7F). Additionally, we assessed the activity of the downstream proteins Smad2/3 and phosphorylated-Smad2/3. The results of Western blot showed that knockdown of circMPP6 inhibited the level of TGF-β and the expression of phosphorylated Smad2 and Smad3 (Fig. 7F). ADAM22 had the same effect as circMPP6 on TGF-β and the phosphorylation levels of Smad2 and Smad3 (Fig. 7G). Of note, pharmacological inhibition of TGF-β signaling with the TGFBR1 inhibitor SB505124 completely abolished circMPP6-induced malignant phenotypes (Fig. 7H-K), demonstrating that ADAM22 promotes tumor progression through activation of the TGF-β/Smad pathway.

### CAF-derived exosomal circMPP6, packaged via hnRNPA2B1, promotes HGSOC progression by targeting ADAM22

**Figure 8:**
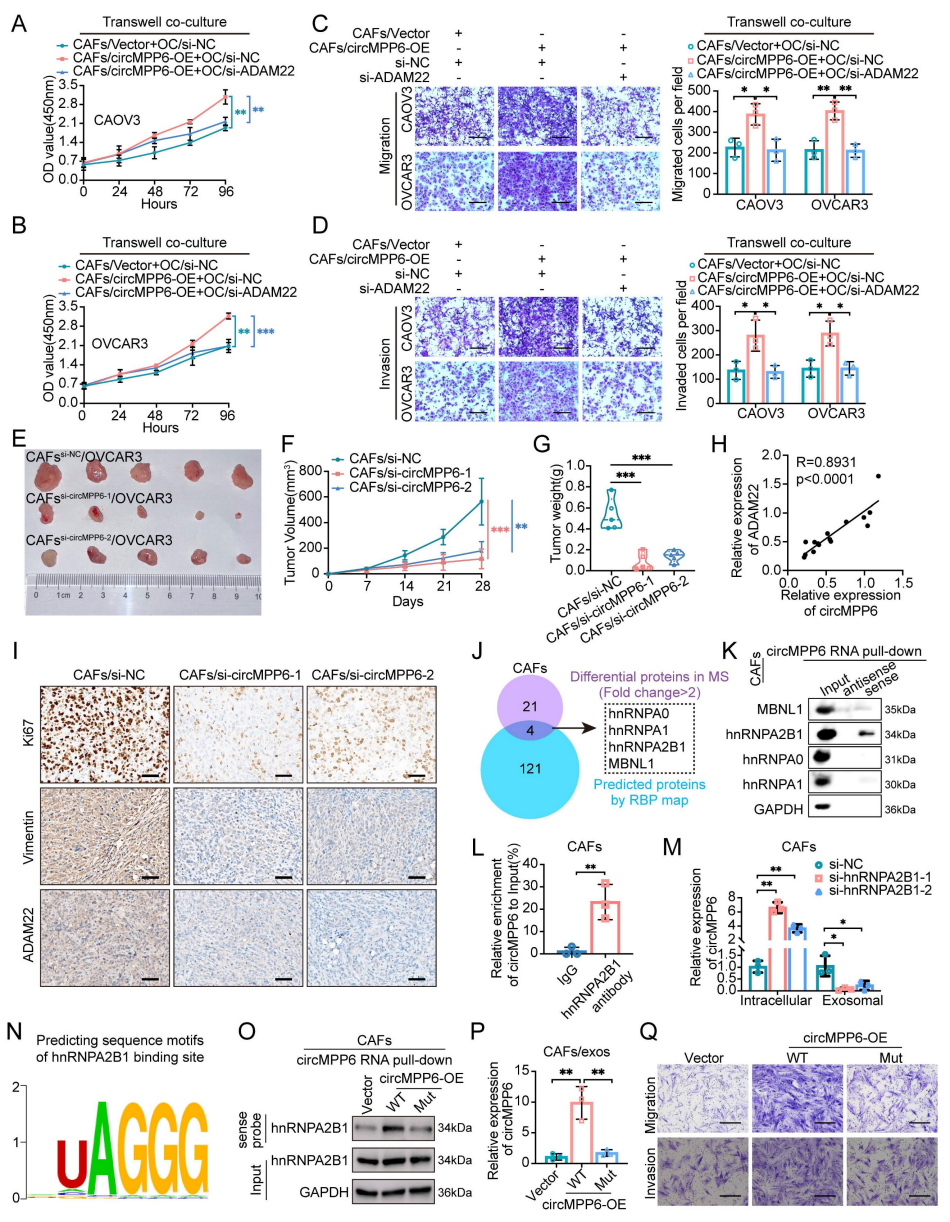
ADAM22 mediates CAFs-derived circMPP6 effects on HGSOC cell viability and metastasis both *in vitro* and *in vivo*. (**A-D**) HGSOC cells transfected with si-ADAM22 or control were co-cultured with CAFs transfected with circMPP6 overexpression (OE) or vector plasmids for 72 hours. After co-culture, viability of CAOV3 (A) and OVCAR3 (B) cells was assessed using the CCK-8 assay. Transwell migration (C) and invasion (D) assays were conducted. Scale bar: 300 μm. (**E-G**) Tumor images (E), tumor volumes (F) and tumor weights (G)of xenograft tumors from co-transplantation of OVCAR3 cells with CAFs with or without circMPP6 knockdown. (**H**) RT-qPCR showed a positive correlation between circMPP6 and ADAM22 expression. (**I**) Immunohistochemistry analysis Ki67, Vimentin, and ADAM22 in xenograft tumors. (**J**) Venn diagram showing the overlap results of the differentially expressed proteins in MS and circMPP6 targets in RBPmap. (**K**) Proteins pulled down by circMPP6 sense or antisense probe were detected by western blot of CAFs. (**L**) Enriched circMPP6 using hnRNPA2B1 antibody was detected by qRT-PCR after RIP assay of CAFs. Data were calculated as input %. (**M**) Intracellular and exosomal circMPP6 expression were detected respectively by qRT-PCR after knockdown of hnRNPA2B1 in CAFs. (**N**) hnRNPA2B1-binding motif predicted by RBPsuite. (**O**) hnRNPA2B1 pulled down by circMPP6 sense probe was detected by western blot after mutating of the 437-441 nt region of circMPP6 in CAFs. (**P**) Exosomal circMPP6 expression was detected by qRT-PCR after mutating of the 437-441 nt region of circMPP6 in CAFs. (**Q**) Cellular migration and invasion were detected by Transwell assay after mutating of the 437-441 nt region of circMPP6 in CAFs. *p < 0.05, **p < 0.01, ***p < 0.001.

To investigate the tumor-promoting role of exosome-packaged circMPP6, co-culture assays were conducted. ADAM22 knockdown partially or completely reversed the increase in proliferation, migration, and invasion observed in ovarian cancer cells co-cultured with circMPP6-overexpressing CAFs (Fig. 8A-D).

Moreover, we assessed whether altering circMPP6 expression in CAFs could affect tumor growth in mouse xenografts co-transplanted with OVCAR3 cells. Remarkably, xenografts from OVCAR3 cells with circMPP6-knockdown CAFs exhibited significantly lower growth rates than those from control CAFs (Fig. 8E-G and Supplementary Fig. S7A). Tissue PCR revealed reduced ADAM22 expression following circMPP6 knockdown (Supplementary Fig. S7B), confirming a linear correlation between ADAM22 and circMPP6 expression (Fig. 8H). Ki67 and vimentin analyses revealed that co-transplantation with circMPP6-knockdown CAFs led to substantially reduced tumor growth and invasion, along with significantly decreased ADAM22 protein expression levels (Fig. 8I and Supplementary Fig. S7C-E).

Given the ability of circMPP6 in CAF exosomes to promote HGSOC metastasis, we explored its intracellular mechanism in parental cells before packaging into exosomes. We performed mass spectrometry (MS) analysis after RNA pull-down in CAFs to investigate the protein-binding role of circMPP6 (Supplementary Fig. S7F). Notably, the RBPmap software predicted 125 potential binding proteins, with an overlap of four proteins, including Heterogeneous Nuclear Ribonucleoprotein A1 (hnRNPA1), Heterogeneous Nuclear Ribonucleoprotein A0 (hnRNPA0), Heterogeneous Nuclear Ribonucleoprotein A2/B1 (hnRNPA2B1), and Muscleblind Like Splicing Regulator 1 (MBNL1) between the experimental and predictive results (Fig. 8J). RNA pull-down assays confirmed that hnRNPA2B1 was the definitive protein that bound to circMPP6 (Fig. 8K). Additionally, the specific binding of hnRNPA2B1 to circMPP6 was verified by RNA immunoprecipitation (RIP) using an hnRNPA2B1 antibody from CAF cell lysates (Fig. 8L). We further evaluated the effect of hnRNPA2B1 knockdown on circMPP6 sorting into exosomes. Knockdown of hnRNPA2B1 in CAFs transfected with hnRNPA2B1 siRNAs (Supplementary Fig. S7G), resulted in a significant increase in circMPP6 expression in cells but a decrease in exosomes (Fig.8M). RBPsuite prediction identified a putative hnRNPA2B1-bingding UAGGG motif at nucleotides 437-441 of circMPP6 (Fig. 8N). Mutating of this region markedly reduced hnRNPA2B1 binding and abolished efficient exosomal loading of circMPP6 (Fig. 8O and 8P), demonstrating that hnRNPA2B1 directly recognizes circMPP6 and mediates its selective exosomal sorting. Next, intracellular functional assays of circMPP6 were conducted. Consistently, the mutant circMPP6 lacking this motif also lost its ability to promote CAF proliferation, migration, and invasion (Figures 8Q and Supplementary Fig. S7H-I), indicating that this sequence is required for both hnRNPA2B1 binding and circMPP6 function.

**Figure 9:**
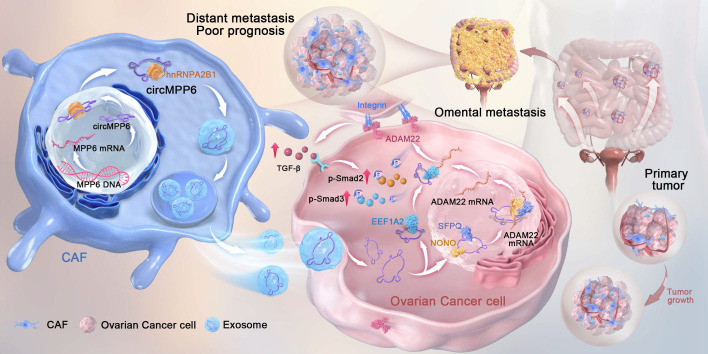
Mechanism illustration: CAF-derived exosomal circMPP6 is mediated by hnRNPA2B1 in CAFs and drives ovarian cancer metastasis by coordinating nuclear and cytoplasmic regulation of ADAM22 to activate TGF-β/Smad signaling in ovarian cancer cells.

In summary, our findings demonstrated that circMPP6 derived from CAFs is bound by hnRNPA2B1 and packaged into exosomes, through which it is transferred to ovarian cancer cells and serves as a crucial regulator of ovarian cancer progression. It enhances cell proliferation, migration, and invasion by interacting with EEF1A2, SFPQ, and NONO, ultimately modulating ADAM22 protein levels and activating the TGF-β signaling pathway (Fig. 9).

## Discussion

Our study revealed that circMPP6 derived from CAFs plays a critical role in ovarian cancer progression. Specifically, we show that circMPP6, sorted into exosomes via interaction with hnRNPA2B1 is transferred from CAFs to HGSOC cells, where it promotes cell proliferation, migration, and invasion by binding to the proteins EEF1A2, SFPQ, and NONO, ultimately enhancing ADAM22 protein levels and activating the TGF-β/Smad signaling pathway. These findings underscore the role of circMPP6 as a pro-tumorigenic factor and suggest that it could serve as a novel prognostic marker and therapeutic target in ovarian cancer.

CircRNAs are characterized by their stable and tissue-specific expression and are gaining attention as potential biomarkers and therapeutic targets across multiple cancer types, including ovarian cancer 36-39. Previous studies have documented dysregulated circRNA profiles in ovarian cancer, with some showing an association with metastasis to the greater omentum 40-42. Here, we found that circMPP6 was significantly upregulated in metastatic omental tissues from patients with HGSOC. Notably, high circMPP6 expression correlated with worse patient outcomes. CAF-derived circRNAs in exosomes have been shown to influence tumor biology by modulating intercellular communication 15, 18, 19, 42. Our data further support this concept, as circMPP6 was expressed prominently in CAFs and localized to areas within HGSOC tumor tissue near CAFs, but was minimally expressed in regions distant from CAFs. This pattern suggests a local paracrine effect, in which CAFs potentially transfer circMPP6 into neighboring cancer cells to promote tumor progression. The functional impact of circMPP6 downregulation in CAFs was demonstrated using both *in vitro* and *in vivo* models, where reduced circMPP6 levels inhibited the proliferation, migration, and invasion of both CAFs and HGSOC cells. These findings suggested that CAF-derived circMPP6 is essential for maintaining the aggressive phenotype of HGSOC cells. Exosomal circMPP6 from CAFs accumulated more in HGSOC cells than circMPP6 from normal fibroblasts (NFs), indicating a selective enrichment mechanism that could enhance intercellular transfer within the tumor microenvironment.

In HGSOC cells, we observed that circMPP6 knockdown suppressed cell proliferation, migration, and invasion, whereas its overexpression promoted these malignant behaviors, reinforcing its oncogenic role in ovarian cancer metastasis. CircRNAs often function through ceRNA networks, acting as molecular sponges for microRNAs 40, 42-49. However, we did not observe any interaction between circMPP6 and AGO2, suggesting that it may operate through alternative regulatory mechanisms in HGSOC cells. Our exploration of protein interactions revealed that circMPP6 binds to EEF1A2 and VARS in the cytoplasm and to SFPQ and NONO in the nucleus. EEF1A2, EEF1B, EEF1D, EEF1G and VARS interact to form elongation factor complex components for protein synthesis 21, 50. Although VARS did not enrich circMPP6 as effectively as EEF1A2, the latter has been extensively studied as an oncogenic protein across various cancers 23, 51, 52, including lung adenocarcinoma, colorectal cancer, and hepatocellular carcinoma, where it promotes tumor growth and metastasis 23, 51, 53-56. Here, we demonstrated that the association of circMPP6 with EEF1A2 in the cytoplasm of HGSOC cells facilitated oncogenic signaling, thus supporting circMPP6's role in ovarian cancer progression. In the nucleus, circMPP6's interaction with SFPQ and NONO enhances the formation of the SFPQ-NONO complex, which is a key player in RNA processing and gene regulation. SFPQ and NONO, members of the *Drosophila Behavioral Human splicing* (DBHS) family, form paraspeckles that regulate diverse nuclear processes, including mRNA splicing and stability 57-62. Notably, both SFPQ and NONO have been implicated in various cancers, including ovarian cancer, where they regulate alternative splicing and apoptotic response to chemotherapy 29, 31, 63-68. We found that circMPP6 upregulates the expression of ADAM22, an oncogene previously unstudied in ovarian cancer, by stabilizing the SFPQ-NONO complex and promoting mRNA stability. ADAM22, a member of the ADAM family, is known to be implicated in tumor progression through interaction with integrins and other signaling pathways 32, 69-72. To our knowledge, this is the first study to report the role in ovarian cancer, establishing ADAM22 as a potential new therapeutic target. Further investigations into how the SFPQ-NONO heterodimer and EEF1A2 specifically regulate ADAM22 mRNA processing and maturation in HGSOC are required. Additionally, our data suggest that EEF1A2 positively influences ADAM22 expression at both mRNA and protein levels, a relationship that warrants a more detailed mechanistic study. *In vivo* and *in vitro* experiments validated the role of ADAM22 in enhancing ovarian cancer cell proliferation, migration, and invasion, confirming its function in circMPP6-mediated oncogenic signaling.

Many members of the ADAMs family promote cell proliferation and invasion by binding to integrin proteins and activating integrin-related signaling pathways 73, 74. ADAM22 also has been reported to promote cancer progression by binding to integrins in pituitary adenoma 32 and head and neck squamous cell carcinoma 75. Our results show that ADAM22 binds to ITGB1 in HGSOC cells. Integrin proteins were verified to activate TGF-β signaling, and involved in a variety of physiological and pathological processes, including tumor survival, development, and re-shaping the tumor microenvironment 33, 34, 76. In gastric cancer, the binding of transforming growth factor β1 (TGF-β1) to ITGB1, regulates downstream signaling molecules, and supports lymph node metastasis 35. In the current study, we demonstrate that circMPP6/ADAM22/ITGB1 enhances TGF-β/Smad signaling-mediated HGSOC metastasis. Notably, there is abundant evidence showing that the TGF-β/Smad signaling pathway is associated with the metastasis of various human cancers 77-80. However, the role of circRNAs in the TGF-β/Smad signaling pathway remains largely unknown. Unravelling the underlying mechanism by which circRNAs regulate the TGF-β pathway may provide a new direction for developing therapeutic agents. The TGF-β/Smad signaling is also widely involved in a variety of biological functions, including cell proliferation, differentiation, communication, extracellular matrix remodeling, angiogenesis and immune evasion 80-83. Here, our results that circMPP6 induces TGF-β1/Smad signaling indicating that it may be involved in these biological processes, which warrant future investigation. Our data revealed a novel mechanism by which circMPP6 enhanced TGF-β and the phosphorylation of SMAD2 and SMAD3 by facilitating ADAM22. Targeting circRNAs may be a novel strategy against aberrant TGF-β signaling activation in cancer.

Regarding the mechanism of action in CAFs, circMPP6 interacted with hnRNPA2B1 to be loaded circMPP6 into exosomes derived from CAFs. Specifically, nucleotides 437-441 of circMPP6 are critical for its interaction with hnRNPA2B1, and are indispensable for hnRNPA2B1-mediated sorting of circMPP6 into exosomes. Our results are consistent with those of several previous studies showing that hnRNPA2B1 plays a role in the packaging of non-coding RNA into exosomes in multiple types of tumors 84-86. In ovarian cancer, hnRNPA2B1 selectively packages metastasis-promoting ncRNAs into exosomes, thus facilitating ovarian carcinoma metastasis 85. To our knowledge, this is the first study to illustrate the novel role of hnRNPA2B1 in CAFs of HGSOC. Therefore, our findings reveal a novel mechanism in which hnRNPA2B1-mediated loading of circMPP6 into CAFs secreted exosomes could provide new strategies for developing targeted therapies against omental metastasis in HGSOC.In conclusion, our findings highlight CAF-derived circMPP6 as a mediator of ovarian cancer metastasis and poor prognosis. CAF-derived circMPP6 is sorted into exosomes via interaction with hnRNPA2B1. Subsequently, exosome-packaged circMPP6 was internalized by HGSOC cells, promoting cancer cell proliferation and invasion by engaging in cytoplasmic and nuclear pathways involving EEF1A2, SFPQ, and NONO to facilitate ADAM22 expression, ultimately enhancing TGF-β/Smad signaling in HGSOC. This study reveals a novel mechanism by which CAF-specific circRNAs contribute to the aggressive behavior of ovarian cancer cells, providing new insights into prognosis and potential personalized treatment strategies for HGSOC. Elucidating the circMPP6/ADAM22/TGF-β/Smad signaling axis may pave the way for developing novel therapeutic strategies and improving prognostic prediction of cancer progression. Further investigations of circMPP6 and its associated pathways may advance the development of targeted therapies for patients with ovarian cancer.

## Methods

### Clinical sample collection

This study received approval from the Ethics Committee of Women's Hospital, School of Medicine, Zhejiang University (approval number: IRB-20210122-R). Informed consent was obtained from each patient prior surgery. None of the patients had undergone chemotherapy or radiotherapy before surgery. All fresh tissue samples, pathologically confirmed as HGSOC, were immediately preserved in liquid nitrogen upon collection. Information on the patients with paraffin-embedded tissue samples for FISH assays is provided in Supplementary Table S1.

### RNA extraction and RNA sequencing

Total RNA from tissue or cell samples was extracted using TRIzol Reagent (Invitrogen, USA). The integrity of RNA was assessed, and samples with an RNA Integrity Number ≥ 7 were used for library construction. Sequencing was conducted on an illumina NovaSeq 6000 by NovelBio Co., Ltd (China), following the manufacturer's instructions.

### RNase R and RT-qPCR analysis

For RNase R treatment, RNA samples were incubated with or without 2 U/μg RNase R (Epicentre Technologies) at 37 °C for 15 minutes. RT-qPCR analysis was performed according to previously described protocols 87, with primer sequences listed in Supplementary Table S2.

### Fluorescence in situ hybridization (FISH) and immunofluorescence assay

Cy3-labeled probes targeting the back-splice junction of circMPP6 was designed by RiboBio (Guangzhou, China). FISH assays were carried out per the Ribo™ Fluorescent *In Situ* Hybridization Kit (RiboBio, China) instructions. For immunofluorescence assays, cells were blocked with 3% bovine serum albumin (BSA) for 1 hour, then incubated with primary antibodies overnight at 4 °C and with secondary antibodies at room temperature for 1 hour. After washing, cells were counterstained with DAPI to visualize nuclei and observed under a confocal laser scanning microscope. The percentages of positive cells were quantified using Image J.

### SiRNAs and plasmids transfection

siRNAs were synthesized by TsingKe (Hangzhou, China). Transient transfection was conducted using DharmaFECT Transfection Reagents (Thermo, USA) per the standard protocol. Target sequences of siRNAs are listed in Supplementary Table S3. Overexpression plasmids for circMPP6 were constructed by Calm Biotechnology (Shanghai, China). 3×Flag-tagged NONO wide-type and NONO RRM domain-truncated plasmids were synthesized by Genechem Biotech (Shanghai, China). Plasmids were transfected using X-treme GENE HP DNA Transfection Reagent (Roche, China).

### CCK-8 cell growth assay and cell apoptosis analysis

Cell growth was assessed using Cell Counting Kit 8 assays (Dojindo, Japan). Cell apoptosis in CAFs were evaluated by flow cytometry using Annexin V-FITC and propidium iodide kit (Multisiences, China), following the manufacturer's protocol.

### Transwell migration and invasion assays

Transwell chambers (8 μm pore size, Falcon, USA) were used to assess cell invasion and migration abilities. A total of 2 × 10^5^ cells were seeded in the upper chamber with OPTI-MEM (Gibco) medium, with (for invasion) or without (for migration) Matrigel (Corning, USA), and 20% FBS medium was added to the lower chamber. After 12-24 hours, non-invasive or non-migratory cells were removed, while penetrated cells were fixed, stained, and counted.

### Coculture system

CAFs and NFs were isolated from HGSOC omental metastatic tissues and normal omental tissues, respectively, following established protocols 88. Transfected CAFs were seeded in the upper chamber of a 12-well Transwell plate (0.4 μm, LABSELECT, China), while ovarian cancer cells were seeded in the lower chambers. After 72 hours, ovarian cancer cells were used for subsequent functional assays.

### Exosome purification, characterization, quantitation and treatment *in vitro* and *in vivo*

For exosome isolation from cells, after reaching 90% confluence, cells were washed three times with PBS and incubated with conditioned medium containing exosome-depleted FBS for 72 hours. The supernatant was collected and purified by differential ultracentrifugation as previously described 89. For exosome extraction from tissues, samples were cut into pieces less than 2 mm in diameter, then dissociated in FBS-free DMEM medium containing type I collagenase (1 mg/mL) and type I DNase (0.2 mg/mL) at 37 ℃ for 40 minutes. The lysate was centrifuged at 3000 rpm for 10 minutes twice, and the supernatant was filtered through a 70μm filter before exosome purification as per established protocols 90.

Exosome samples were visualized with transmission electron microscopy (JEM-1230, JEOL, Japan) at 80 kV 89. The particle size distribution was analyzed using ZetaView, following instructions. Exosome markers CD63 and CD81 were used for characterization. Exosome concentrations were quantified by the BCA Protein Assay Kit (Beyotime, China). For cell treatment, 50 μg exosomes in 1 mL culture medium were applied to CAOV3 or OVCAR3 cells for 72 hours. For* in vivo* treatments, each mouse received 20 μg exosomes per 100 μl PBS via intraperitoneally injected twice a week.

### PKH26-labelled exosome transfer

Purified exosomes were labeled with PKH26 (Sigma-Aldrich, USA) as per the manufacturer's protocol, followed by 0.5% BSA quenching. The mixture was filtrated through a 0.22 μm filter to remove unbound dye, and exosomes were precipitated using the ExoQuick Exosome Precipitation Kit (SBI, USA). CAOV3 or OVCAR3 cells were incubated with PKH26-labeled exosomes for 24 hours, fixed with 4% paraformaldehyde, and then counterstained with FITC Phalloidin for cell skeleton and DAPI for nuclei visualization. Exosome uptake was observed by confocal laser scanning microscopy.

### Western blot and immunohistochemistry

For western blot analysis, proteins were separated on 4-12% YoungPAGE gels (GenScript, USA) and transferred onto 0.22 μm PVDF membranes (Bio-Rad, USA) using the eBlot L1 protein transfer system (GenScript, USA). Membranes were blocked with 5% milk, incubated with primary antibodies overnight, followed by secondary antibody incubation, and visualized using the Fdbio-Dura Enhanced Chemiluminescence Kit. For immunohistochemistry, formalin-fixed and paraffin-embedded samples were deparaffinized, rehydrated, washed in PBS, and antigen retrieval was performed in 0.01 M sodium citrate buffer (pH 6.0) at 100 °C for 15 minutes. Antibodies used are listed in Supplementary Table S4. For IHC analyses, Ki67 positivity was quantified as the percentage of positive cells using Image J. For other proteins, IHC scores was determined by multiplying the proportion score (0-25%, 26-50%, 51-75%, 76-100%) and staining intensity score (negative, weak, moderate, strong). At least five representative fields per section were analyzed, and mean values were used for statistical comparison.

### RNA pull-down

Biotin-labeled RNA probes (Supplementary Table S5) were synthesized by Genepharma (China). RNA pull-down assays were performed using the Pierce™ Magnetic RNA-Protein Pull-down Kit (Thermo Scientific). Streptavidin magnetic beads were incubated with probes for 30 minutes at room temperature, washed twice, then incubated with cell lysates in RNA-protein binding buffer at 4 °C overnight. After washing, proteins attached to beads were eluted for mass spectrometry (MS) analysis (Lumingbio, Shanghai, China) and western blotting.

### RNA immunoprecipitation (RIP)

The RIP assay was conducted with the EZ-Magna RIP kit (Millipore, USA) as per the manufacturer's instructions. Protein A/G magnetic beads were incubated with antibodies for 30 minutes at room temperature, then washed with RIP wash buffer. Cell lysates were incubated with beads overnight at 4°C, and co-precipitated RNA was extracted and analyzed by qRT-PCR.

### Co-immunoprecipitation (co-IP)

Cells were lysed with IP lysis buffer (Thermo Scientific) and incubated with 5 μg primary antibody in a rotating incubator overnight at 4 °C, using IgG as the negative control. PierceTM Protein A/G Magnetic Beads (Thermo Fisher Scientific, USA) were then added and rotated for 2 hours at room temperature. Eluted proteins were analyzed by western blotting.

### Nude mouse xenograft experiment

Animal experiments were approved by the Animal Ethical and Welfare Committee of Zhejiang Chinese Medical University (approval number: IACUC-20230313-18). Three-week-old female BALB/c nude mice were obtained from Shanghai Silaike Laboratory Animal Co, Ltd. (China). Ovarian cancer xenografts were established by subcutaneous injection of CAOV3 or OVCAR3 cells (2 × 10^6^ cells of 100μl PBS) and CAFs or NFs cells (1 × 10^6^ cells of 100μl PBS) into the left armpit of the mice. Prior to injection, CAFs were transfected with si-circMPP6-1, si-circMPP6-2, or si-NC. Tumor volumes were measured weekly using the formula: Tumor volume (mm^3^) = (length×width^2^)/2. For metastatic models, luciferase-expressing OVCAR3 cells (the same amount) were injected intraperitoneally. Mice were randomly assigned to three groups and treated with different exosomes or PBS*.* For imaging, mice received an intraperitoneal injection of 150 mg/kg D-luciferin (Yeasen, Shanghai, China) and were imaged 15 minutes later using an IVIS Lumina LT system. Regions of interest (ROI) were analyzed using LIVING IMAGE software. After euthanizing the mice, tissues were dissected, fixed in 4% paraformaldehyde, embedded in paraffin, and used for H&E staining or IHC analysis.

### Statistical analysis

Data were analyzed using GraphPad Prism 9.0 (GraphPad Software, USA) and are presented as mean ± standard deviation (SD). A Student's t test was applied for comparisons between two normally distributed groups, while the Mann-Whitney tests was used otherwise. For comparisons among more than two groups, ANOVA was applied. Each experiment was repeated at least three times, with p-values < 0.05 was considered statistically significant.

## Supplementary Material

Supplementary figures and tables.

## Data Availability

The datasets used and/or analyzed during the current study are available from the corresponding author on reasonable request.
