# Supplementary material for: CAF-derived exosomal circMPP6 drives ovarian cancer metastasis by coordinating nuclear and cytoplasmic regulation of ADAM22 to activate TGF-β/Smad signaling
Source: Int J Biol Sci. 2026 Mar 30;22(8):3969–89. doi: 10.7150/ijbs.126013 (PMC13137859; doi:10.7150/ijbs.126013)
Supplement: Supplementary file 1 — Supplementary figures and tables. [file ijbsv22p3969s1.pdf]

Supplementary Figures

Figure S1

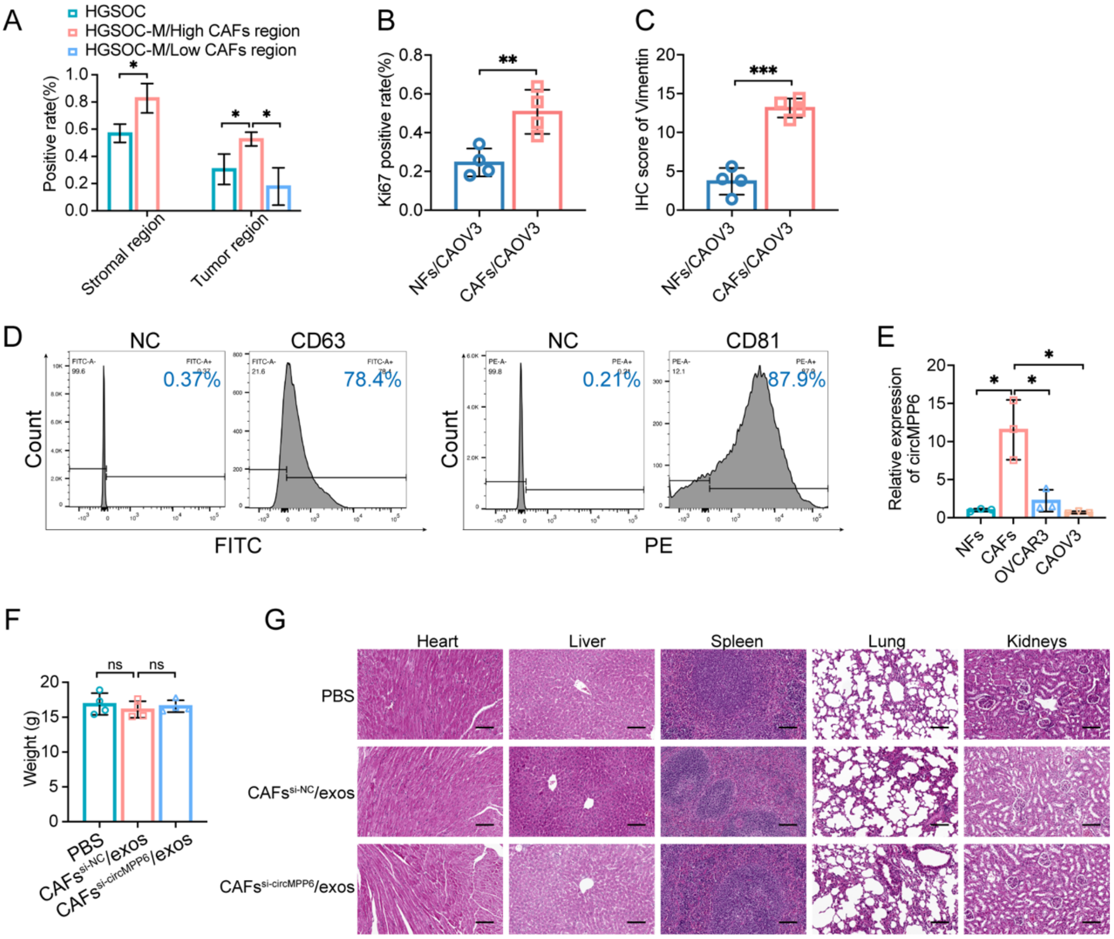

Supplementary Figure S1. (**A**) Quantification of the rate of circMPP6-positive cells in stromal region and tumor region based on FISH analysis of circMPP6 expression in primary and metastatic HGSOC tissues. (**B**) The average Ki-67 positive rate in xenograft tumors is presented as the mean value plus the standard deviation (SD) based on IHC analysis of Ki67 in each treatment group. (**C**) The relative protein expression of vimentin in xenograft tumors is depicted as the mean value plus the SD based on IHC analysis of vimentin in each treatment group. (**D**) Flow Cytometry of CD63, CD81 in the exosomes fraction. (**E**) RT-qPCR of circMPP6 in NFs, CAFs, CAOV3, and OVCAR3 cells. (**F**) Mouse weights at sacrifice. (**G**) The preliminary toxicity analysis of indicated exosomes in mice bearing OVCAR3-derived xenograft tumors. Representative images of heart, liver, spleen, lung and kidney harvested from mice in each group were shown.

Figure S2

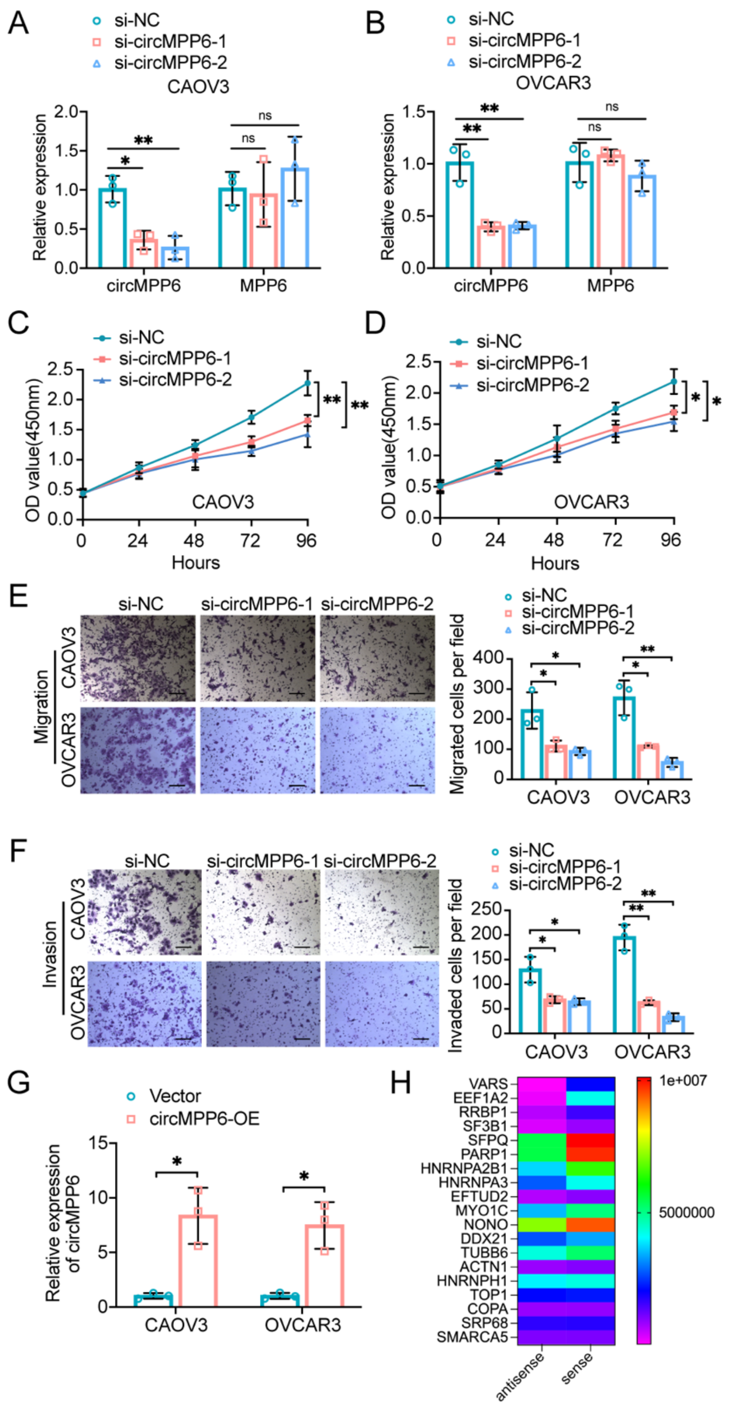

Supplementary Figure S2. related to Figure 4. (**A-B**) Expression levels of circMPP6 and MPP6 mRNA in CAOV3 cells (**A**) and OVCAR3 cells (**B**) were detected by qRT-PCR after transfection with two si-circMPP6 backsplicing-specific siRNAs or a negative control siRNA. (**C-D**) CAOV3 cells (**C**) and OVCAR3 cells (**D**) transfected with circMPP6 siRNAs or control analyzed for proliferation by CCK-8. (**E-F**) Migration (E) and invasion (F) assays for HGSOC cells transfected with circMPP6 siRNAs or control. (**G**) Expression levels of circMPP6 in CAOV3 and OVCAR3

cells were detected by qRT-PCR after transfection with circMPP6 plasmid or a negative control plasmid. (**H**) Protein profile obtained from protein mass spectrometry analysis after RNA pull-down in CAOV3 cells.

Figure S3

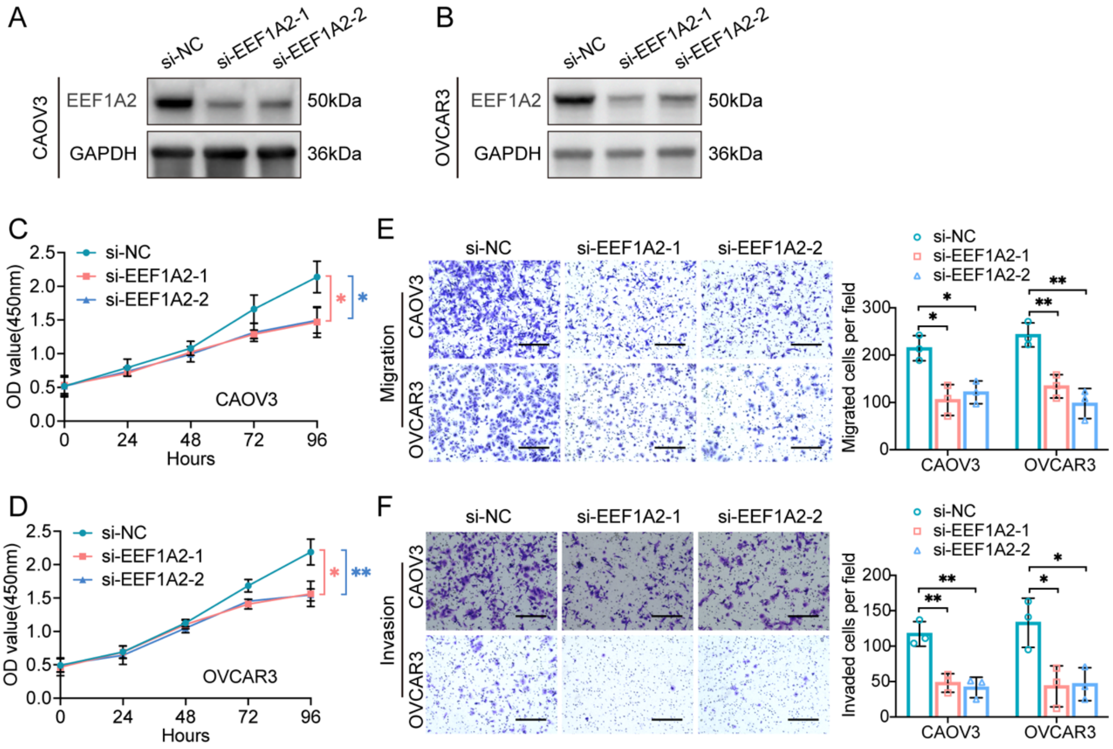

Supplementary Figure S3. related to Figure 4. (**A-B**) CAOV3 (**A**) and OVCAR3 (**B**) cells were transfected with two EEF1A2 siRNAs or negative control. EEF1A2 expression was determined by immunoblotting analysis. (**C-F**) Cellular proliferation (**C-D**), migration (**E**) and invasion (**F**) were detected by CCK-8 assay and transwell assay.

Figure S4

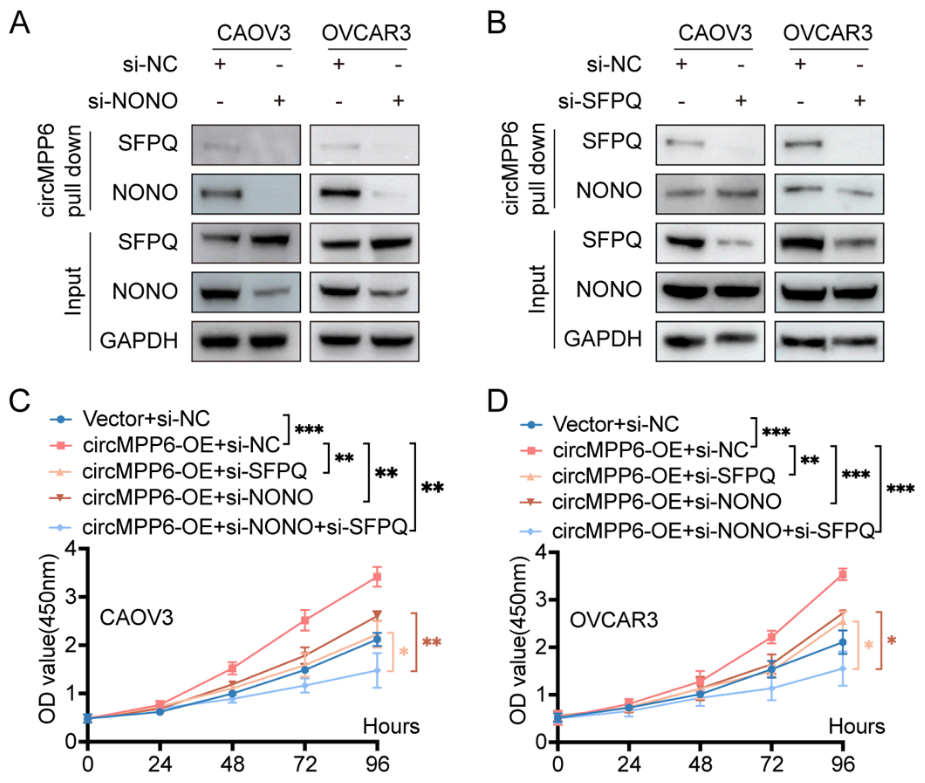

Supplementary Figure S4. related to Figure 5. (**A**) Western blot validation of pulled proteins with biotin-labeled circMPP6 probes in CAOV3 or OVCAR3 cells transfected with NONO siRNAs or negative control. (**B**) Western blot validation of pulled proteins with biotin-labeled circMPP6 probes in CAOV3 or OVCAR3 cells transfected with SFPQ siRNAs or negative control. (**C-D**) HGSOC cells were transfected with circMPP6 plasmid, circMPP6 plasmid plus si-SFPQ, circMPP6 plasmid plus si-NONO, circMPP6 plasmid plus si-NONO plus si-SFPQ, and negative control, respectively. CCK-8 assays were performed to determine the proliferation changes of CAOV3 (**C**) and OVCAR3 (**D**) cells.

Figure S5

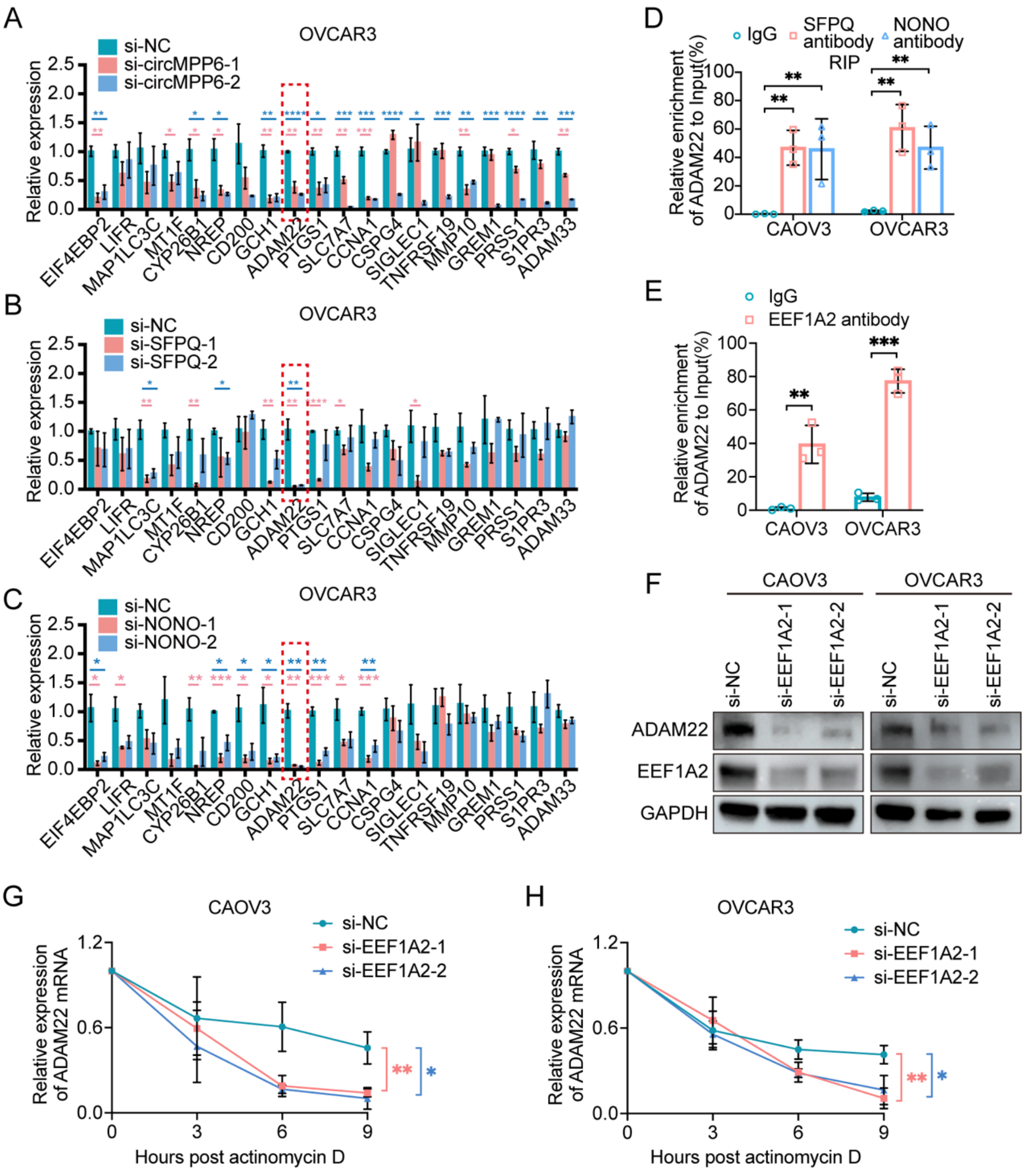

Supplementary Figure S5. related to Figure 6. (**A**) RT-qPCR analysis of differentially expressed mRNAs in OVCAR3 cells transfected with two circMPP6 siRNAs or negative control. (**B**) RT-qPCR analysis of differentially expressed mRNAs in OVCAR3 cells transfected with two SFPQ siRNAs or negative control. (**C**) RT-qPCR analysis of differentially expressed mRNAs in OVCAR3 cells transfected with two NONO siRNAs or negative control. **(D)** RT-qPCR of ADAM22 mRNA in SFPQ or NONO RIP assays. (**E**) ADAM22 mRNA recruited by EEF1A2 antibody or IgG from the lysates in CAOV3 and OVCAR3 cells was detected by RIP assay followed by qRT-PCR. (**F**) Protein levels of ADAM22 and EEF1A2 in CAOV3 and OVCAR3 cells detected by

immunoblotting. (**G-H**) ADAM22 mRNA expression in NONO or SFPQ knockdown CAOV3(G) and OVCAR3(H) cells treated with actinomycin D.

Figure S6

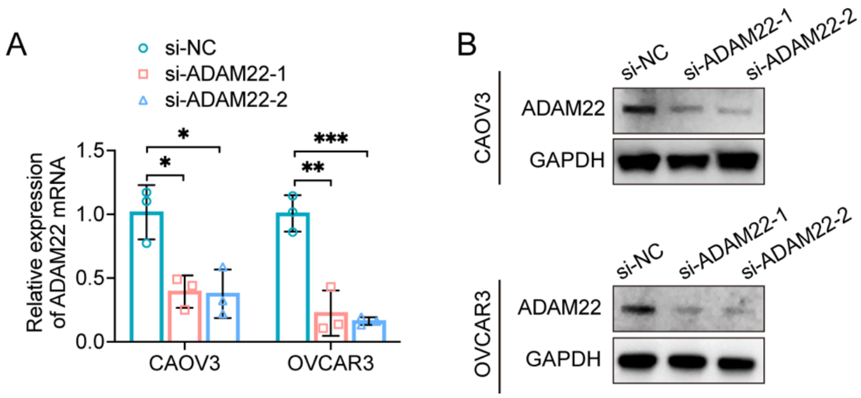

Supplementary Figure S6. related to Figure 7. (**A-B**) CAOV3 and OVCAR3 cells were transfected with two ADAM22 siRNAs or negative control. ADAM22 expression was determined by RT-qPCR (**A**) and immunoblotting analysis (**B**).

Figure S7

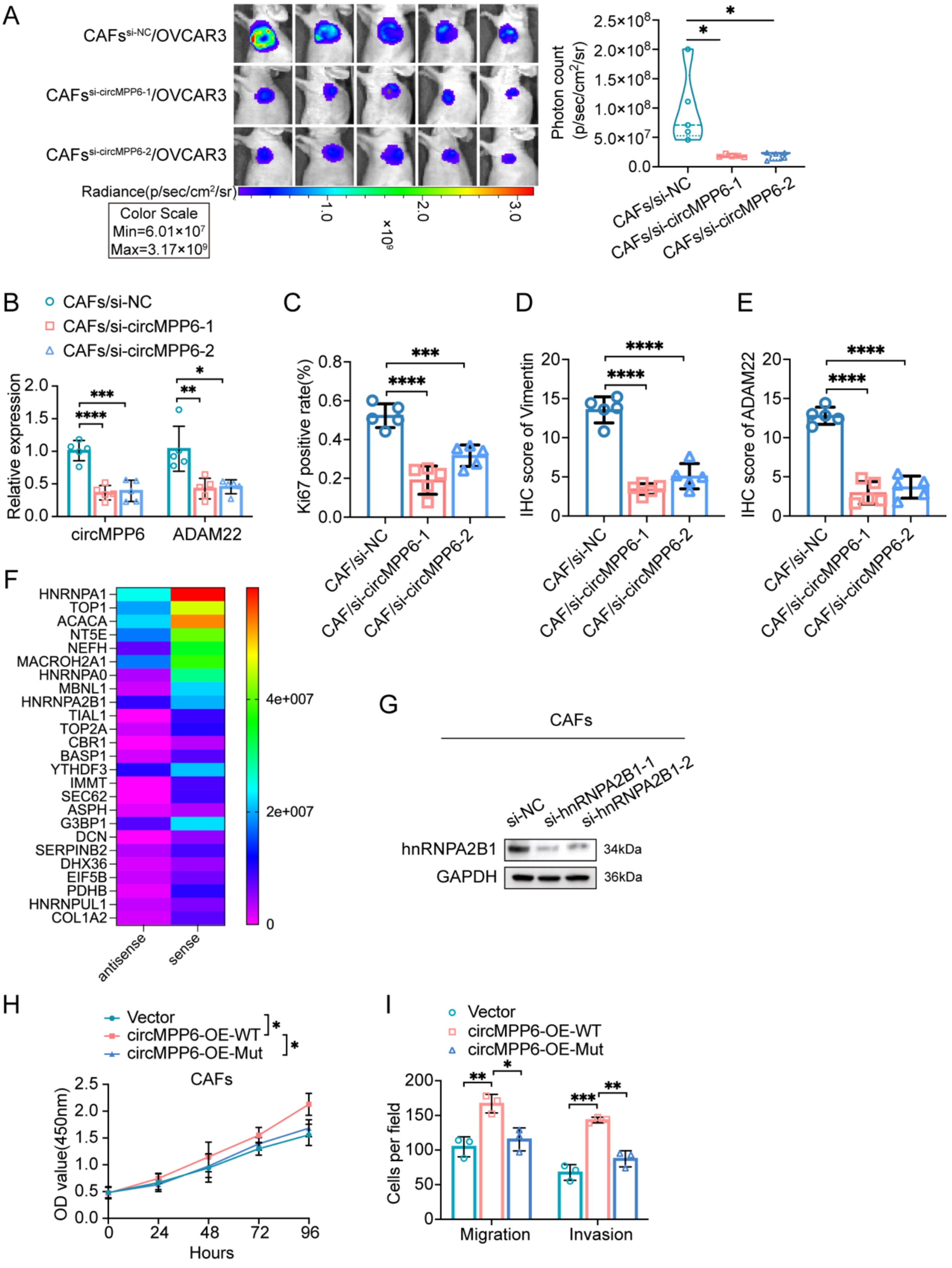

Supplementary Figure S7. related to Figure 8. (**A**) IVIS images of xenograft tumors from co-transplantation of OVCAR3 cells with CAFs with or without circMPP6 knockdown. (**B**) RT-qPCR measured circMPP6 and ADAM22 mRNA levels in tumors. (**C**) The average Ki-67 positive rate in each treatment group is presented as the mean value plus the standard deviation (SD) based on IHC

analysis of Ki67 in various groups of transplanted tumors. (**D**) The relative protein expression of vimentin in each treatment group is depicted as the mean value plus the SD based on IHC analysis of vimentin in various groups of transplanted tumors. (**E**) The relative protein expression of ADAM22 in each treatment group is depicted as the mean value plus the SD based on IHC analysis of ADAM22 in various groups of transplanted tumors. (**F**) Protein profile obtained from protein mass spectrometry analysis after RNA pull-down in CAF cells. (**G**) Whole cell lysates of CAFs were extracted to detect the expression of the hnRNPA2B1 and GAPDH proteins. (**H**) Cellular proliferation was detected by CCK-8 assay. (**I**) Cellular migration and invasion were detected by Transwell assay.

Supplementary Tables

Table S1 Clinicopathologic characteristics of high grade serous ovarian cancer associated with circMPP6 expression for FISH analysis.

Case No.	Age	Gender	FIGO stage	Time to overall survival (months)	Time to recurrence(months)
1	52	female	IIIC	26	20
2	61	female	IIIC	19	14
3	49	female	IIIC	45	21
4	69	female	IV	7	5
5	58	female	IIIC	52	11
6	66	female	IIIC	60	18
7	51	female	IIIA	60	34
8	55	female	IIIC	60	33
9	52	female	IIIC	60	21
10	63	female	IIIC	60	60
11	45	female	IIIC	60	60
12	65	female	IIIC	60	60
13	43	female	IIIC	60	24
14	49	female	IIIC	60	13
15	49	female	IIIA2	60	60
16	50	female	IIIC	60	39
17	50	female	IIIc	60	18
18	55	female	IIIc	60	60
19	55	female	IIIc	60	19
20	64	female	IIIc	60	60
21	59	female	IIIb	60	60
22	57	female	IIIC	60	12
23	47	female	IIIC	60	16
24	47	female	IIIC	60	60

25	57	female	IIIA	60	60
26	47	female	IIIc	60	60
27	56	female	IIIc	60	60
28	59	female	IIIc	60	60
29	52	female	IV	60	16
30	54	female	IIB	60	22
31	41	female	IIIc	60	60
32	52	female	lllc	24	13
33	45	female	IIIB	31	13
34	62	female	IIIC	30	19
35	71	female	IIB	24	11
36	52	female	IIIC	23	10
37	78	female	IIIC	20	14
38	49	female	IIIA1	41	36
39	69	female	IIIc	17	7
40	62	female	IIIc	18	8
41	48	female	IIIC	19	10
42	51	female	IIIB	32	22
43	63	female	IIIC	19	12
44	45	female	IIIB	5	3
45	64	female	IIIC	17	9
46	61	female	IIIC	60	60
47	50	female	IIIC	60	48
48	54	female	IIIC	60	17
49	66	female	IIIC	29	14
50	77	female	IIIC	60	12

Table S2 Sequences of primers used for PCR in this study.

Item	Sequence
circMPP6 (divergent)	Forward: 5'- TCAGAGTTTATGCCCTATGTG -3'
	Reverse: 5'- TAGGTCTATTTCTTCTGCTCC-3'
linearMPP6 (convergent)	Forward: 5'- TCTGGTAATTGCCCGAATCCT -3'
	Reverse: 5'- AGATTTTTAGGGTGACACTTCCAC -3'
GAPDH	Forward: 5'- TCACCACCATGGAGAAGGC -3'
	Reverse: 5'- GCTAAGCAGTTGGTGGTGCA -3'
18s rRNA	Forward: 5'- TTAATTCCGATAACGAACGAGA -3'
	Reverse: 5'- CGCTGAGCCAGTCAGTGTAG -3'
circMPP6	Forward: 5'- TCAGAGTTTATGCCCTATGTG -3'
	Reverse: 5'- TAGGTCTATTTCTTCTGCTCC -3'
circUBAP2	Forward: 5'- GAGAGCTTGCACCACCAAAA -3'
	Reverse: 5'- CTGAGGCTTGACTGTGAGGA -3'
circPCSK5	Forward: 5'- GCGGGGAGTCCTACGATAAG -3'
	Reverse: 5'- TTGCTTCAACCATGTCCGTG -3'
circZFAT	Forward: 5'- GACGGTGACTGTGGTTAAGC -3'
	Reverse: 5'- TTCTTCGGCCTCTGTGATGT -3'
circSEC31A	Forward: 5'- TTGCTTCCTCTCCACTTCGT -3'
	Reverse: 5'- TGATATCTTCTGGCGGCTGT -3'
circNAPEPLD	Forward: 5'- ACCAGCATGTAGACCCAGAA -3'
	Reverse: 5'- TCCGTGCTGAATTTTGACGT -3'
circSTK3	Forward: 5'- GCAACCTCACCACAAGTACA -3'
	Reverse: 5'- GTTGGTGGTGGATTTGTGGG -3'
circRICTOR	Forward: 5'- AGAGGACACAAGCACTTCGA -3'
	Reverse: 5'- TCCTCATAGTGAAAGCCCAGT -3'
linearMPP6	Forward: 5'- TCTGGTAATTGCCCGAATCCT-3'
	Reverse: 5'- AGATTTTTAGGGTGACACTTCCAC-3'
EEF1A2	Forward: 5'- GTCAAGGAAGTCAGCGCCTAC-3'
	Reverse: 5'- TGAACCACGGCATGTTGGG-3'
SFPQ	Forward: 5'- AGCGATGTCGGTTGTTTGTTG-3'
	Reverse: 5'- AGCGAACTCGAAGCTGTCTAC-3'
NONO	Forward: 5'- CTAGCGGAGATTGCCAAAGTG-3'

	Reverse: 5'- GTTCGTTGGACACATACTGAGG-3'
EIF4EBP2	Forward: 5'- TAGCCCTGGCACCTTAATTGA-3'
	Reverse: 5'- ATCCCCAACTGCATGTTTCCT-3'
LIFR	Forward: 5'- TGGAACGACAGGGGTTCAGT-3'
	Reverse: 5'- GAGTTGTGTTGTGGGTCACTAA-3'
MAP1LC3C	Forward: 5'- GAGCCACGGAAGCCTTTTACT-3'
	Reverse: 5'- TGGGAGGCGTAGGTCATGT-3'
MT1F	Forward: 5'- CCCACTGCTTCTTCGCTTCT-3'
	Reverse: 5'- AGGAGCAGCAGCTCTTCTTG-3'
CYP26B1	Forward: 5'- GGCAACGTGTTCAAGACGC-3'
	Reverse: 5'- TGCTCGCCCATGAGGATCT-3'
NREP	Forward: 5'- GTCTGGGTCAGTCAAGAACCA-3'
	Reverse: 5'- AGGCAGCGTTTGTCTCATCG-3'
CD200	Forward: 5'- AAGTGGTGACCCAGGATGAAA-3'
	Reverse: 5'- AGGTGATGGTTGAGTTTTGGAG-3'
GCH1	Forward: 5'- GTGAGCATCACTTGGTTCCAT-3'
	Reverse: 5'- GTAAGGCGCTCCTGAACTTGT-3'
ADAM22	Forward: 5'- TTGGAACGTCATTCATTCTCGAT-3'
	Reverse: 5'- TTCCTCGGATATGGCCCTGG-3'
PTGS1	Forward: 5'- CGCCAGTGAATCCCTGTTGTT-3'
	Reverse: 5'- AAGGTGGCATTGACAAACTCC-3'
SLC7A7	Forward: 5'- CCCAAGGGTGTGCTCATATACA-3'
	Reverse: 5'- CCAGTTCCGCATAACAAAGGG-3'
CCNA1	Forward: 5'- ACATGGATGAACTAGAGCAGGG-3'
	Reverse: 5'- GAGTGTGCCGGTGTCTACTT-3'
CSPG4	Forward: 5'- CTTTGACCCTGACTATGTTGGC-3'
	Reverse: 5'- TGCAGGCGTCCAGAGTAGA-3'
SIGLEC1	Forward: 5'- CCTCGGGGAGGAACATCCTT-3'
	Reverse: 5'- AGGCGTACCCCATCCTTGA-3'
TNFRSF19	Forward: 5'- CCAGCAAGGTCAACCTCGT-3'
	Reverse: 5'- CAGAGCCGTTGTACTGAATGT-3'
NEGR1	Forward: 5'- GGGAGGTGATAAGTGGTCAGT-3'
	Reverse: 5'- CTGGGTGTATGTTGAGTCTGAAC-3'
GREM1	Forward: 5'- CGGAGCGCAAATACCTGAAG-3'
	Reverse: 5'- GGTTGATGATGGTGCGACTGT-3'

PRSS1	Forward: 5’- AGCCAGGCTAAGTGTGAAGC-3’
	Reverse: 5’- AATCACCCTGACATGAATCCTTG-3’
S1PR3	Forward: 5’- CGGCATCGCTTACAAGGTCAA-3’
	Reverse: 5’- GCCACGAACATACTGCCCT-3’
ADAM33	Forward: 5’- CTGCTCTGGCCAGTGCCAGG-3’
	Reverse: 5’- GCACCACTGGCTGCCCATCTG-3’

Table S3 Sequences of siRNAs against specific targets in this study.

Item	Sequence
circMPP6 siRNA-1	sense: 5’- ACCAAGCUUCUGACCCAAU(dT)(dT)-3’
	antisense: 5’- AUUGGGUCAGAAGCUUGGU(dT)(dT)-3’
circMPP6 siRNA-2	sense: 5’- GACCCAAUGCAGCAAGUCU(dT)(dT)-3’
	antisense: 5’-AGACUUGCUGCAUUGGGUC(dT)(dT)-3’
EEF1A2 siRNA-1	sense: 5’- GGACCAUUGAGAAGUUCGA(dT)(dT)-3’
	antisense: 5’- UCGAACUUCUCAAUGGUCC(dT)(dT) -3’
EEF1A2 siRNA-2	sense: 5’-GCGACAACGUCGGCUUCAA(dT)(dT)-3’
	antisense: 5’- UUGAAGCCGACGUUGUCGC(dT)(dT)-3’
NONO siRNA-1	sense: 5’- CCAGCAAUUUCACAAGGAA(dT)(dT) -3’
	antisense: 5’- UUCCUUGUGAAAUUGCUGG(dT)(dT) -3’
NONO siRNA-2	sense: 5’- CAGGCGAAGUCUUCAUUCA(dT)(dT) -3’
	antisense: 5’-UGAAUGAAGACUUCGCCUG(dT)(dT)-3’
SFPQ siRNA-1	sense: 5’-GGAAGAACUUCACAAUCAA(dT)(dT) -3’
	antisense: 5’-UUGAUUGUGAAGUUCUUCC(dT)(dT)-3’
SFPQ siRNA-2	sense: 5’- GCAUAGGUUAUGAAGCUAA(dT)(dT) -3’
	antisense: 5’- UUAGCUUCAUAACCUAUGC(dT)(dT) -3’
ADAM22 siRNA-1	sense: 5’- GGCGACUGACAACAAGUUU(dT)(dT) -3’
	antisense: 5’-AAACUUGUUGUCAGUCGCC(dT)(dT) -3’
ADAM22 siRNA-2	sense: 5’- GGAUAGGUUCUGAUUGCAA(dT)(dT) -3’
	antisense: 5’-UUGCAAUCAGAACCUAUCC(dT)(dT) -3’

Table S4 Antibodies used in this study.

Antigens	Manufacturer	Application
CD81	SBI, USA	1:1000 for WB
CD63	SBI, USA	1:1000 for WB
GAPDH	Diagbio, China	1:2000 for WB
TGF-β	Proteintech, China	1:1000 for WB
p-smad2	Abclonal, China	1:1000 for WB
Smad2	Abclonal, China	1:1000 for WB
p-Smad3	Abclonal, China	1:1000 for WB
Smad3	Abclonal, China	1:1000 for WB
hnRNPA2B1	Proteintech, China	1:1000 for WB<br>5 μg per reaction for RIP
hnRNPA1	Abclonal, China	1:1000 for WB
hnRNPA0	Abclonal, China	1:1000 for WB
MBNL1	Abclonal, China	1:1000 for WB
EEF1A2	Proteintech, China	1:1000 for WB<br>5 μg per reaction for RIP
SFPQ	Proteintech, China	1:1000 for WB<br>5 μg per reaction for RIP
NONO	Proteintech, China	1:1000 for WB<br>5 μg per reaction for RIP
VARS	Proteintech, China	1:1000 for WB<br>5 μg per reaction for RIP
ADAM22	Abcam, USA	1:1000 for WB<br>1:100 for IHC
SMA	Abcam, USA	1:500 for IF
FAP	Abclonal, China	1:500 for IF
Vimentin	Abcam, USA	1:500 for IF

		1:400 for IHC
Ki67	Proteintech, China	1:400 for IHC

Table S5 Sequences of probes used for RNA pull down in this study.

**Iterm**	**sequence**
circMPP6 sense probe	5’-AAGACTTGCTGCATTGGGTCAGAAGCTTG-3’Biotin
circMPP6 antisense probe	5’-ACCAAGCTTCTGACCCAATGCA-3’Biotin
